# Investigation of the Neurotoxicity Mechanisms of Ni^2+^ in Rat Neocortical Neurons Through Transcriptome Analysis

**DOI:** 10.3390/ijms26094014

**Published:** 2025-04-24

**Authors:** Chen Meng, Yang Lu, Yan Huang, Xiaoying Lü

**Affiliations:** State Key Laboratory of Digital Medical Engineering, Southeast University, Nanjing 210096, China; vince.mengchen@hotmail.com (C.M.); 220222299@seu.edu.cn (Y.L.); hylucky@seu.edu.cn (Y.H.)

**Keywords:** neuron, Ni^2+^, neurotoxicity, cytotoxicity, excitability, transcriptome

## Abstract

The cytotoxic effects of Ni^2+^ released from nickel-based alloy implants on tissues have been a longstanding research focus in biocompatibility studies. However, investigations into the neurotoxicity of Ni^2+^ remain relatively limited. Building on our previous findings that Ni^2+^ can rapidly affect the excitability of neuronal networks, this study further investigated the neurotoxic effects of prolonged Ni^2+^ exposure. First, the cytotoxicity effects of Ni^2+^ on rat neocortical neurons in vitro were evaluated by MTT cell viability assay, and changes in the length of the axon initial segment of neurons caused by Ni^2+^ exposure were quantified. Next, transcriptome sequencing was employed to identify differentially expressed genes (DEGs) induced by Ni^2+^ treatment, and four DEGs—*Hk2*, *Ldha*, *Cd9*, and *Nfasc*—were selected for qRT-PCR validation. The ATP content of neurons was measured to assess cellular energy metabolism under Ni^2+^ influence. Finally, by comparing these experimental results with our previous findings, this study explored the neurotoxicity mechanisms of Ni^2+^ and analyzed the correlation between its neurotoxicity and cytotoxicity. This study revealed that the neurotoxicity mechanisms of Ni^2+^ are associated with the concentration of Ni^2+^ and the duration of its action. When at low concentrations or with short exposure times, Ni^2+^ suppresses the excitability of the neuronal networks by rapidly blocking Ca^2+^ channels, whereas at high concentrations or with prolonged exposure, it further inhibits the network’s excitability by activating the HIF-1α pathway and inducing obvious cytotoxicity.

## 1. Introduction

Nickel-based alloys and nickel-containing stainless steel are extensively utilized in cardiovascular implantable medical devices. In the human physiological environment, these implants may corrode and release water-soluble Ni^2+^ [1,2,3]. The cytotoxic effects of Ni^2+^ have long been a focal point in biocompatibility research, with numerous studies investigating its cytotoxicity and underlying mechanisms [4,5,6]. Our group has previously conducted a comprehensive analysis of the cytotoxicity of Ni^2+^ on L929 cells [7,8,9]. With the advancement of interventional surgery techniques, nickel-based alloy implants have increasingly been adopted in neurosurgery, particularly for treating cerebral vascular malformations [2]. The existing literature indicates that the local tissue surrounding nickel-based alloy implants exhibits significantly higher nickel concentrations compared to other regions [10]. Clinical studies have also reported neurological deficits, such as epilepsy, limb weakness, and language disorders, in some patients treated for cerebral aneurysms using nickel-based alloy implants. These adverse effects are likely associated with the impact of Ni^2+^ released from the implants on the surrounding neurons [11,12,13,14,15].

The toxic effects of chemical substances on organisms can be categorized based on their endpoints of action into carcinogenicity, genotoxicity, neurotoxicity, cytotoxicity, reproductive and developmental toxicity, oxidative stress, dermal toxicity, immunotoxicity, and ocular toxicity [16]. Cytotoxicity is defined as chemical potency that induces changes in cellular functions and leads to cell death. In in vitro biocompatibility tests, cytotoxicity is a commonly used toxicity evaluation metric. Cytotoxicity is typically assessed by evaluating changes in cell viability, cell growth, and specific parameters of cell metabolism [16,17,18]. In contrast to cytotoxicity, neurotoxicity refers to the adverse effects of chemical substances on the structure and function of the nervous system, with a primary focus on alterations in neural functions that are directly related to the electrophysiological properties of neurons and neuronal networks [16,19]. For neurons, due to their unique electrophysiological properties and functions, the toxic effects of chemicals cannot be fully assessed solely through cytotoxicity assays. Instead, a comprehensive evaluation requires integrating methods from both electrophysiology and cell biology to achieve a thorough understanding. Currently, not only do the international standards for the in vitro biocompatibility testing of implantable devices lack specific evaluation methods for neurotoxicity, but also the literature on biocompatibility in relation to neurons lacks research on neurotoxicity and investigations into the relationship between cytotoxicity and neurotoxicity [18]. Several years ago, our group began to study the cytotoxicity and neurotoxicity of chemicals and their interrelationship by combining cytological and electrophysiological methods. In previous studies on the influence of silver nanoparticles (AgNPs) on neuronal networks, we found that at a low concentration of 5 μM, AgNPs began to affect the electrical excitability of neuronal networks, demonstrating neurotoxicity without causing cytotoxicity (cell viability > 90%). When the concentration was increased to 100 μM, AgNPs exhibited neurotoxicity within 4 h; however, after 12 h, they showed both significant cytotoxicity (cell viability < 80%) and neurotoxicity [20]. These findings suggest that the neurotoxic effects of chemicals on neurons may precede their cytotoxic effects.

Ni^2+^ is a common Ca^2+^ channel blocker in neuroscience research. It can directly and rapidly influence the electrophysiological properties of neurons, thereby exhibiting neurotoxic effects [21,22,23,24,25]. In the field of biocompatibility research, current studies on the neurotoxicity of Ni^2+^ are relatively limited, and no research has specifically investigated the potential interrelationship between its cytotoxicity and neurotoxicity mechanisms. In this study, we first evaluated the cytotoxicity of Ni^2+^ through an MTT assay. Next, we analyzed changes in the electrophysiological properties of neurons exposed to Ni^2+^ by investigating the alterations in the length of the axon initial segment (AIS). Finally, we examined changes in gene expression of neurons following Ni^2+^ exposure, and integrated these findings with previous research on the effects of Ni^2+^ on neuronal network excitability, thereby conducting a preliminary investigation into the mechanisms underlying these changes and their interrelationships.

## 2. Results

### 2.1. The Impact of Ni^2+^ on the Cell Viability of Neurons

**Figure 1:**
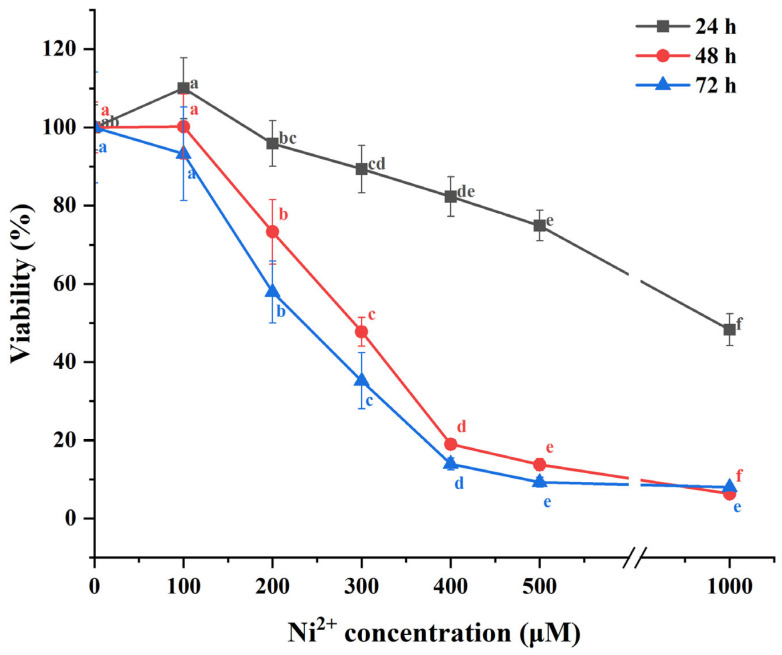
The mean cell viability of neurons exposed to different concentrations of Ni^2+^ for 24, 48, or 72 h (*n* = 6; means that do not share a letter are significantly different).

In this study, the MTT assay was employed to assess the cell viability of neocortical neurons exposed to varying concentrations of Ni^2+^ for 24, 48, and 72 h. The cytotoxic effects of Ni^2+^ on neurons were evaluated by analyzing changes in cell viability. Cytotoxicity was indicated when the cell viability ≤ 80%. In the experiment, one-way ANOVA followed by Tukey’s post hoc test were used to analyze and perform multiple comparisons for the data from each group after 24 h of Ni^2+^ treatment. For the data from each group after 48 or 72 h of Ni^2+^ treatment, Welch’s ANOVA analysis was conducted using the Welch ANOVA application (v1.00) in Origin 2025, followed by multiple comparisons using the Games–Howell post hoc test. The results are shown in Figure 1 (Tables S1–S3 in the Supplementary Materials). A total of 100 μM Ni^2+^ did not show significant cytotoxicity on neurons. However, after 48 and 72 h of treatment with 200 μM Ni^2+^, Ni^2+^ began to exhibit significant cytotoxicity, with cell viability decreasing to 73.30 ± 8.22% and 57.95 ± 7.93%, respectively. As the concentration of Ni^2+^ increased, its cytotoxicity gradually intensified, leading to a continued decline in cell viability. This result is consistent with the findings from our previous study on Ni^2+^ cytotoxicity in L929 cells [7,8]. After Ni^2+^ exposure for 48 or 72 h, the changes in the cell viability of neurons were most pronounced within the concentration range of 100–500 μM. Therefore, we selected 48 h as the exposure duration and 100, 200, and 500 μM as the concentrations for subsequent experiments.

### 2.2. The Effect of Ni^2+^ on the AIS Length of Neocortical Neurons

**Figure 2:**
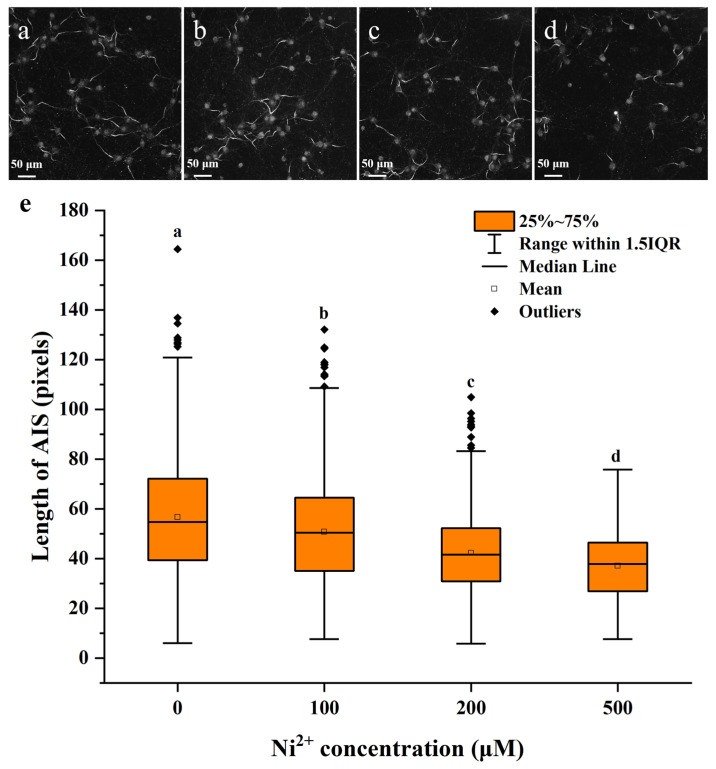
The axon initial segment (AIS) length of neurons exposed to different concentrations of Ni^2+^ for 48 h. (**a**–**d**) Representative images of Ankyrin-G staining in the AIS of neurons from the control group and experimental groups treated with 100, 200, or 500 μM Ni^2+^. (**e**) Comparison of the AIS length across the different groups (*n* = 784, *n* = 816, *n* = 823, and *n* = 548 for the 0, 100, 200, and 500 μM Ni^2+^ groups, respectively; data that do not share a letter are significantly different).

The AIS is a unique region of neuronal axon, where action potentials are initiated. Consequently, neuronal excitability is intricately linked to the AIS properties, and alterations in its length and position significantly influence neuronal function and play a crucial role in neural plasticity. To date, we have not found any relevant studies reporting the effect of Ni^2+^ on the AIS length. In this study, Ankyrin-G was used to label the AIS via immunofluorescence staining. Following treatment with 100, 200, or 500 μM Ni^2+^ for 48 h, changes in the AIS length in neurons were quantified and are presented in Figure 2. The experimental results (Tables S4 and S5 in the Supplementary Materials) were analyzed using the Post Hoc Analysis for Nonparametric Tests application (v1.33) in Origin 2025. Data from each group were compared using the Kruskal–Wallis test, followed by the Conover–Iman post hoc test. In the experiment, we observed that the AIS of in vitro cultured neocortical neurons in each experimental group was consistently positioned near the soma. In this situation, the length of the AIS was positively correlated with neuronal excitability [26,27,28]. The results indicated that as Ni^2+^ concentration increased, the AIS length in the neurons significantly decreased, suggesting that Ni^2+^ affected the electrophysiological function of neurons and inhibited their excitability [27,29]. Compared to cell viability, the properties of neuronal AIS are more sensitive to low concentrations of Ni^2+^, indicating that Ni^2+^ neurotoxicity may manifest at lower concentrations than cytotoxicity.

### 2.3. The Influence of Ni^2+^ on Gene Expression in Neocortical Neurons

Through the aforementioned cytological experiments, we confirmed that Ni^2+^ could inhibit neuronal cell viability and exert a substantial impact on their morphology and function. To further elucidate the potential mechanisms underlying these changes, we investigated the effects of Ni^2+^ on neuronal gene expression.

**Figure 3:**
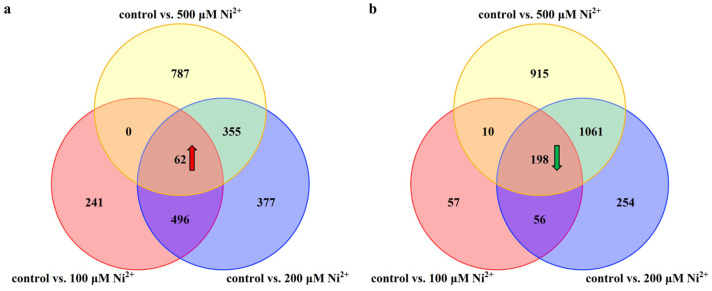
Venn diagrams illustrating differentially expressed genes (DEGs) in each group following 48 h of treatment with varying concentrations of Ni^2+^ (*n* = 3). (**a**) Venn diagram of upregulated genes across different Ni^2+^ concentration treatment groups; (**b**) Venn diagram of downregulated genes across different Ni^2+^ concentration treatment groups.

Firstly, the gene expression profiles of rat neocortical neurons following 48 h of Ni^2+^ exposure were analyzed using transcriptome sequencing. The results indicated that, compared to the control group, the three experimental groups treated with 100, 200, or 500 μM Ni^2+^ exhibited 799, 1290, and 1204 genes as being significantly upregulated, respectively, and 321, 1569, and 2184 genes as being significantly downregulated, respectively. Among these differentially expressed genes (DEGs), a total of 62 genes (Figure 3a) were consistently upregulated and 198 genes (Figure 3b) were consistently downregulated across all Ni^2+^ concentrations after 48 h of treatment, resulting in 260 genes showing consistent expression changes (Tables S6 and S7 in the Supplementary Materials).

**Figure 4:**
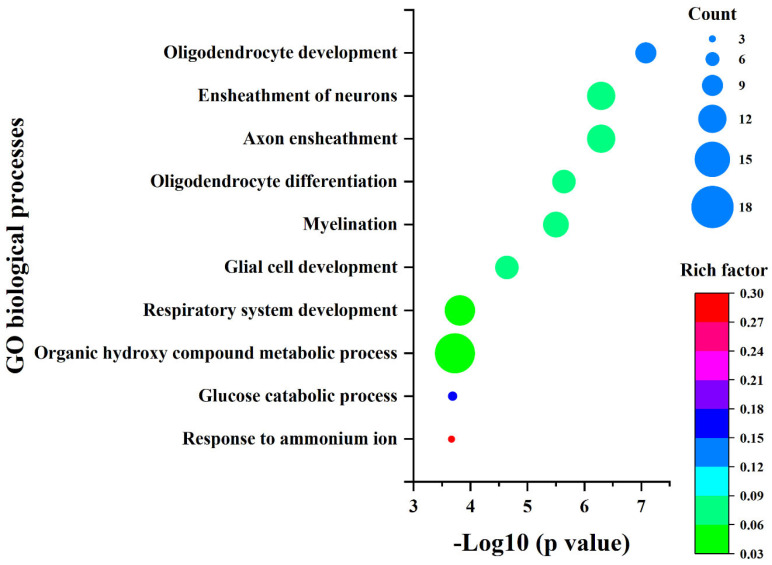
The GO biological process enrichment results for the selected DEGs.

**Figure 5:**
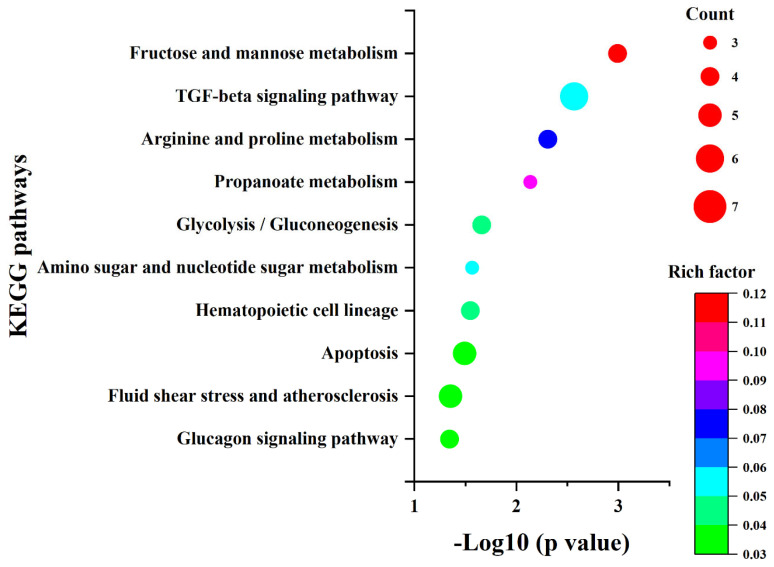
The KEGG pathway enrichment results for the selected DEGs.

**Figure 6:**
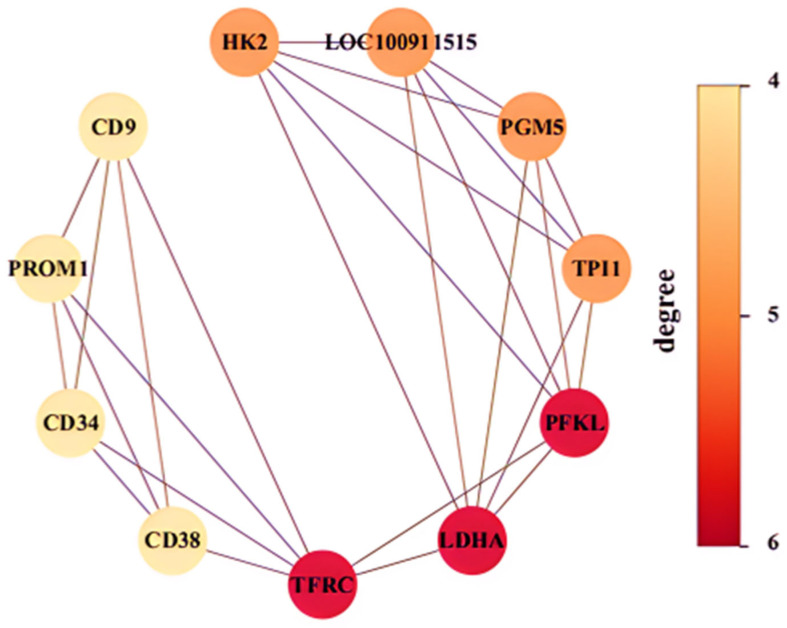
The largest sub-network in the protein–protein interaction network.

The 260 DEGs were analyzed for their GO biological processes and KEGG pathways using the Metascape database [30]. The enrichment results (Tables S8 and S9 in the Supplementary Materials) were sorted by *p*-value from smallest to largest, and the top 10 items are presented in Figure 4 and Figure 5, respectively. Additionally, we constructed the protein–protein interaction network of the proteins encoded by these 260 genes using the STRING database and identified five sub-networks through Cytoscape using MCODE analysis. For this study, we selected the largest sub-network, which contained the most nodes, for detailed analysis (Figure 6) [31,32,33].

In the enrichment results presented in Figure 4 and Figure 5, as well as the sub-network depicted in Figure 6, five genes were consistently identified: *Hk2*, *Pfkl*, *Tpi1*, *Ldha*, and *Cd9*. Among these, *Hk2*, *Pfkl*, *Tpi1*, and *Ldha* encode the key enzymes involved in glycolysis, playing crucial roles in cellular energy metabolism. *Cd9* encodes a protein belonging to the Tetraspanins family, which is known for its significant regulatory functions in cell adhesion, migration, and differentiation [34,35,36,37].

### 2.4. The Effect of Ni^2+^ on ATP Levels in Neocortical Neurons

**Figure 7:**
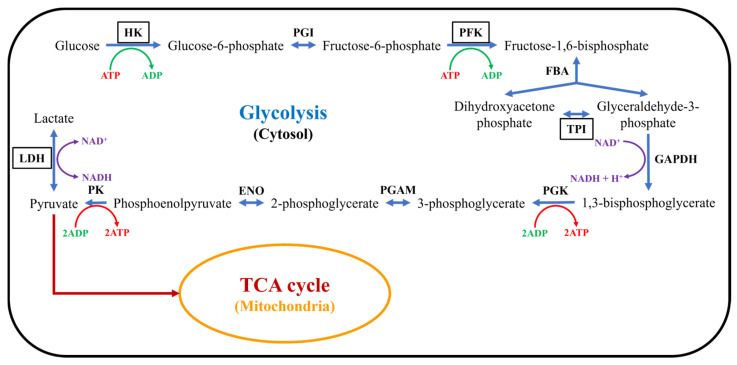
The glycolytic pathway in cellular energy metabolism.

**Figure 8:**
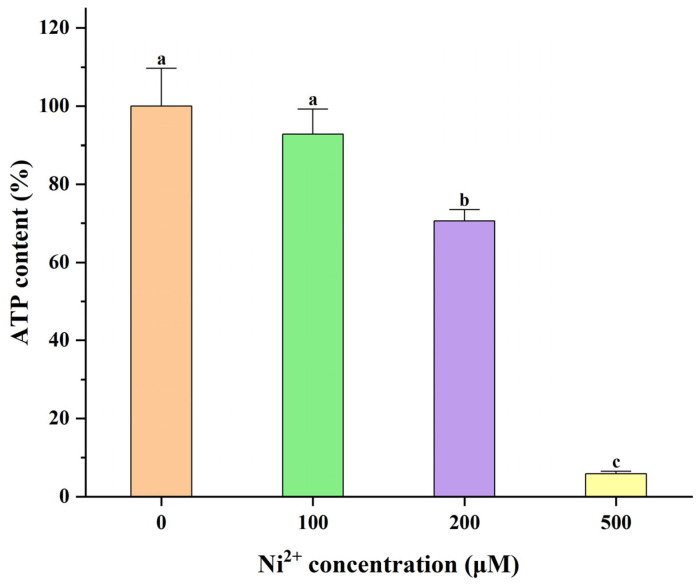
The mean ATP content of neurons exposed to various concentrations of Ni^2+^ for 48 h (*n* = 4; means that do not share a letter are significantly different).

Given that the proteins encoded by *Hk2*, *Pfkl*, *Tpi1*, and *Ldha*, identified through bioinformatics analysis, are all involved in the glycolytic pathway and are closely associated with energy metabolism (Figure 7), this study verified the effect of Ni^2+^ on cellular energy metabolism via ATP content assays [36,37]. The changes in the ATP content of neurons after 48 h of treatment with 100, 200, or 500 μM Ni^2+^ are shown in Figure 8 (Table S10 in the Supplementary Materials). The data from each group were analyzed using the Welch ANOVA application (v1.00) in Origin 2025 via Welch’s ANOVA, followed by the Games–Howell post hoc test. There was no significant difference in ATP content between the 100 μM Ni^2+^ treatment group and the control group without Ni^2+^. However, 200 μM Ni^2+^ significantly reduced the neuronal ATP content to 70.62 ± 2.17% of the control group, while the ATP content in the 500 μM Ni^2+^ treatment group decreased to only 5.82 ± 0.59% of the control group. These results were consistent with those from the MTT assay, indicating that Ni^2+^ concentrations ≥ 200 μM for 48 h significantly inhibited neuronal energy metabolism.

### 2.5. qRT-PCR Analysis

**Figure 9:**
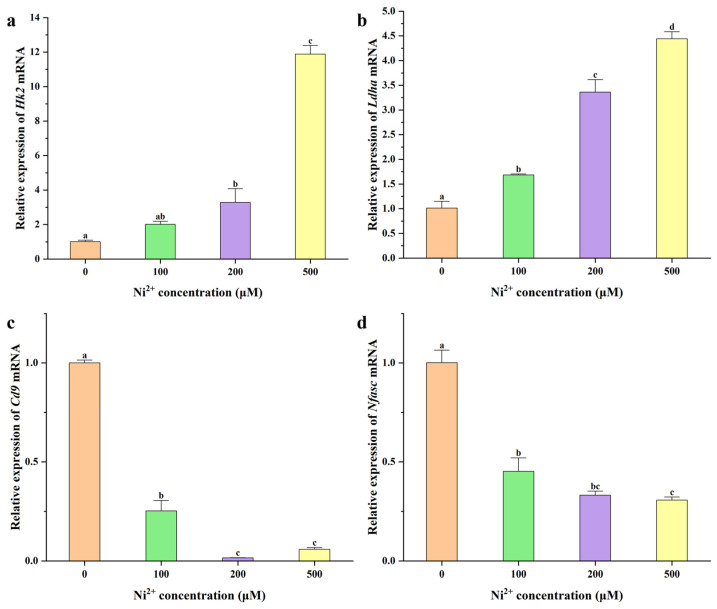
The mRNA expression levels in neocortical neurons after 48 h of exposure to different concentrations of Ni^2+^ (*n* = 3; means that do not share a letter are significantly different). (**a**) *Hk2* expression; (**b**) *Ldha* expression; (**c**) *Cd9* expression; (**d**) *Nfasc* expression.

The protein encoded by *Hk2* catalyzes the formation of glucose-6-phosphate from ATP and glucose in the first step of glycolysis, thereby determining the amount of glucose entering the glycolytic pathway. The protein encoded by *Ldha* catalyzes the conversion of pyruvate to lactate in the final step of glycolysis, allowing pyruvate to exit the mitochondrial TCA cycle and recycling NAD+ to maintain glycolytic flux [35,36,37]. Therefore, *Hk2* and *Ldha* were selected for qRT-PCR analysis to investigate the mechanism by which Ni^2+^ affects neuronal energy metabolism. The protein encoded by *Cd9* is localized in the paranodal regions of axons and plays a critical role in axon development and myelination [38,39]. Additionally, transcriptome sequencing revealed significant changes in the expression levels of the *Nfasc* gene, which encodes a protein essential for maintaining the structure of the AIS. Therefore, *Cd9* and *Nfasc* were selected for qRT-PCR analysis to investigate the mechanisms by which Ni^2+^ influenced the neuronal structure and function. The qRT-PCR results are presented in Figure 9 (Table S11 in the Supplementary Materials).

In rat neocortical neurons, the expression of the *Hk2* gene was significantly elevated after 48 h of exposure to 200 μM Ni^2+^ compared to the control group (Figure 9a). Similarly, the *Ldha* gene expression was significantly increased after 48 h of exposure to 100 μM Ni^2+^ (Figure 9b). Both *Hk2* and *Ldha* expression levels exhibited an upward trend with increasing Ni^2+^ concentrations. The significant upregulation of *Hk2* and *Ldha* suggested that Ni^2+^ markedly enhanced glycolysis in neurons. Glycolysis is an anaerobic metabolic pathway that converts glucose into pyruvate, which can be further converted to lactate under hypoxic conditions. Previous studies have shown that Ni^2+^ activates the hypoxia-inducible factor 1α (HIF-1α) pathway, leading to the upregulation of glycolysis-related genes and a significant increase in HK2 and LDHA protein expression [40,41,42,43,44]. Our findings were consistent with these reports, confirming that Ni^2+^ promotes glycolysis in neocortical neurons by activating the HIF-1α pathway. During glycolysis, pyruvate is converted to lactate, bypassing the mitochondrial TCA cycle, which reduces cellular energy metabolism efficiency and ATP production, ultimately leading to decreased neuronal viability (Figure 1 and Figure 8).

The expression of *Cd9* in neurons was significantly reduced after 48 h of exposure to 100 μM Ni^2+^ compared to the control group. The expression level decreased with increasing Ni^2+^ concentration, reaching its lowest point at 200 μM Ni^2+^, and showed no significant change with further increases in Ni^2+^ concentration (Figure 9c). The reduction in *Cd9* expression led to axonal myelin loss, resulting in abnormal electrophysiological function. This manifested as a decrease in the amplitude of action potentials generated by the AIS and a reduction in the axonal signal transmission speed [34]. While some studies have explored the effects of the HIF-1α pathway on *Cd9* expression under hypoxic conditions, the results are inconsistent, indicating that the HIF-1α pathway is not the sole determinant of *Cd9* expression [45,46,47,48]. Therefore, the specific cause of the significant reduction in *Cd9* expression in neurons following Ni^2+^ treatment remains to be elucidated.

The expression level of *Nfasc* was significantly reduced compared to the control group after 48 h of exposure to 100 μM Ni^2+^, and continued to decrease with increasing Ni^2+^ concentration (Figure 9d). NFASC is a critical protein for maintaining the structure of the AIS in neurons. It functions in concert with Ankyrin-G on the neuronal axon to ensure AIS integrity. A reduction in *Nfasc* expression results in the structural damage of the AIS. Research indicates that Ankyrin-G on the AIS plays a crucial role in anchoring NFASC; its absence can affect the distribution of NFASC along axons, while there is no evidence suggesting this impacts *Nfasc* gene expression [49,50,51]. The mechanism underlying the Ni^2+^-induced downregulation of *Nfasc* gene expression remains unclear.

This study revealed through immunofluorescence staining experiments that Ni^2+^ exposure led to a reduction in Ankyrin-G expression in axons, resulting in a shortened AIS (Figure 2). However, transcriptome analysis showed no significant changes in the expression levels of *Ank3*, the gene encoding Ankyrin-G, across different Ni^2+^ concentrations, indicating that Ni^2+^ primarily affects the Ankyrin-G protein rather than its transcription. Studies have demonstrated that activation of the HIF-1α pathway can elevate cytoplasmic Ca^2+^ levels [52,53,54]. Increased cytoplasmic Ca^2+^ in neurons activates calpain, leading to the hydrolysis of Ankyrin-G and the disruption of the AIS structure, ultimately causing the observed shortening of AIS [55,56].

## 3. Discussion

### 3.1. The Acute and Long-Term Effects of Ni^2+^ on Neurons

In our previous study, we investigated the acute effects of Ni^2+^ exposure on the excitability of the neuronal network in the neocortex region of rat brain slices using MEA. Our results demonstrated that Ni^2+^ significantly inhibits neuronal network excitability within a short exposure period, consistent with the findings from other groups [24,25]. Given the brief exposure duration, this inhibitory effect is primarily attributed to the blockade of Ca^2+^ channels [21,22,23,24,25]. Following Ca^2+^ channel blockage, intracellular Ca^2+^ levels decrease due to the continued activity of intracellular Ca^2+^ pumps. Previous studies have demonstrated that low concentrations of Ni^2+^ can mitigate the increase in intracellular Ca^2+^ content caused by anemia or hypoxia through Ca^2+^ channel blockade, thereby exerting a protective effect on neurons [57,58]. The slightly higher (albeit not significantly) cell viability observed in the 100 μM Ni^2+^ group compared to the control group in the MTT assay after 24 h of Ni^2+^ exposure (Figure 1) may also have been attributed to this protective mechanism.

A critical factor contributing to the increase in intracellular Ca^2+^ during anemia or hypoxia is the activation of the HIF-1α pathway. Both this study and numerous others have experimentally demonstrated that long-term exposure to Ni^2+^ can activate the HIF-1α pathway, leading to an elevation in intracellular Ca^2+^ levels [40,41,42,43,44]. This effect contrasts with the rapid blocking action of Ni^2+^ on Ca^2+^ channels. Although Ni^2+^ is a Ca^2+^ channel blocker, studies have demonstrated that within the concentration range examined in this study, Ni^2+^ primarily blocks T-type Ca^2+^ channels and NMDA receptors, while its effect on L-type Ca^2+^ channels is limited [21,22,23,24]. Notably, L-type Ca^2+^ channels play a critical role in the intracellular Ca^2+^ changes mediated by the HIF-1α pathway [53,59,60]. Therefore, in this study, exposure to Ni^2+^ concentrations of 200 μM or higher for 48 h could have resulted in an increase in intracellular Ca^2+^ levels in neurons, triggering a series of subsequent changes that ultimately led to the inhibition of neuronal network excitability and a reduction in neuronal cell viability [61].

### 3.2. Comparative Analysis of the Effects of Ni^2+^ on Gene Expression in Neurons and L929 Cells

In this study, we analyzed the DEGs in neurons following Ni^2+^ exposure using transcriptome sequencing and compared these with the DEGs in L929 cells after Ni^2+^ treatment, as detected by gene chip analysis in our previous works [7,8]. The results demonstrated that Ni^2+^ had distinct effects on gene expression in the two cell types, particularly in genes associated with the electron transport chain (e.g., *Ndufa2*, *Sdhb*, *Cox6a1*) and focal adhesion pathways (e.g., *Col4a2*, *Fn1*, *Pten*). These genes exhibited significant expression changes in L929 cells but showed no notable alterations in neurons (Table S12 in the Supplementary Materials). Given the significant differences between neurons and other somatic cells in terms of life cycle and functions, the observed gene regulation discrepancies were likely attributable to these distinctions. This suggests that the gene regulation mechanisms in neurons are unique and differ from those in other somatic cells under Ni^2+^ exposure.

**Figure 10:**
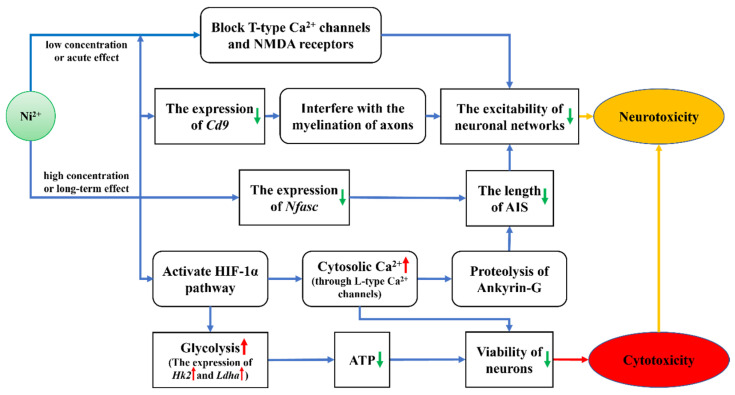
The mechanisms of Ni^2+^ toxicity in rat neocortical neurons.

In summary, based on the research results presented in this study, the mechanisms of Ni^2+^ toxicity in rat neocortical neurons are illustrated in Figure 10. At low concentrations or short exposure times, Ni^2+^ primarily exhibits neurotoxicity by affecting the excitability of the neuronal network without significantly impacting neuronal survival. At high concentrations or prolonged exposure, Ni^2+^ activates the HIF-1α pathway and downregulates the expression of the *Cd9* and *Nfasc* genes, resulting in insufficient cellular energy supply, increased intracellular Ca^2+^ levels, and damage to the AIS. These changes ultimately lead to decreased cell viability and inhibition of neuronal network excitability, manifesting both cytotoxicity and neurotoxicity. These findings indicate that changes in neuronal electrophysiological properties do not necessarily correlate with alterations in cytotoxicity indicators such as cell viability. However, chemical substances that directly affect neuronal survival can negatively impact nervous system function, thereby exhibiting neurotoxic effects. Therefore, the neurotoxic effects of chemical substances on neurons typically precede their cytotoxic effects, consistent with our previous findings on the toxicity of AgNPs in neurons [20].

In the toxicity mechanism of Ni^2+^, changes in intracellular Ca^2+^ content play a significant role. However, due to the fact that commonly used Ca^2+^ fluorescent indicators also label Ni^2+^, and due to the limitations of the experimental conditions, the direct detection of Ni^2+^-induced changes in intracellular Ca^2+^ content in neurons was not feasible in this study [62]. To date, no relevant research reports have been identified. Investigating the effects of Ni^2+^ on intracellular Ca^2+^ levels in neurons is crucial for further understanding its toxicity mechanisms. Additionally, the electrophysiological changes in neurons caused by low concentrations or short exposure times to Ni^2+^ can lead to alterations in gene expression; however, it remains unclear whether these early changes are related to the series of changes observed after prolonged exposure. Extensive studies indicate that the distribution of proteins within neurons significantly impacts their function [26,27,28,39]. Therefore, the neurotoxicity mechanism of Ni^2+^ derived solely from transcriptome analysis in this study is incomplete. Further research is needed to explore changes in protein expression levels and distributions to provide a more comprehensive understanding of the toxicity mechanisms of Ni^2+^ on neurons.

It is widely recognized that research on the biocompatibility of biomaterials can be categorized into two main types: in vitro experiments and in vivo experiments. In vitro experiments offer advantages such as high standardization, excellent reproducibility, relatively low costs, and precise control over experimental conditions; however, they cannot fully replicate in vivo environments. By contrast, in vivo experiments more closely approximate the human physiological environment but are associated with challenges, including extended experimental durations, higher costs, limited control over experimental variables, difficulties in evaluating species-specific differences, and ethical concerns regarding animal welfare. Therefore, in vitro experiments are often prioritized for screening the toxicity of biomaterials and elucidating their mechanisms of action. In contrast to the less well-characterized release and absorption mechanisms of nickel in biological systems, existing studies have demonstrated that nickel-based metallic implants release Ni^2+^ ions under physiological conditions [11]. In in vitro studies examining Ni^2+^ neurotoxicity, it is common practice in both biocompatibility and neuroscience research to use water-soluble nickel salts (e.g., NiCl_2_) as a source of Ni^2+^ [7,8,23,24]. To ensure consistency and comparability with the existing literature, this study employed NiCl_2_ as the Ni^2+^ source and selected the Ni^2+^ concentration as the sole variable. The neurotoxic mechanisms of Ni^2+^ were explored by comparing the results against a blank control group, thereby enhancing the accuracy and reproducibility of the experimental findings.

The aim of this study was to investigate the neurotoxic effects of Ni^2+^ on in vitro cultured neurons using an integrative approach that combined MTT cell viability assays, AIS length analysis, transcriptome sequencing, and qRT-PCR analysis. This integrative strategy provided a more comprehensive understanding of Ni^2+^ effects across multiple biological levels. The integration of transcriptome sequencing for the identification of DEGs and the subsequent validation of selected DEGs via qRT-PCR enhanced the robustness and reliability of the analysis. Compared with the well-documented cytotoxicity of Ni^2+^, current research on the neurotoxicity mechanisms of Ni^2+^ remains relatively limited. This study addressed a critical gap in the scientific literature and offerd valuable insights for future investigations. Nevertheless, it was subject to the inherent limitations of in vitro experimental approaches, which are unable to fully replicate in vivo environments. In the future, conditions permitting, further research could be extended to in vitro organoid models or in vivo animal experiments, thereby facilitating a more comprehensive understanding of Ni^2+^-induced neurotoxicity.

## 4. Materials and Methods

### 4.1. The Isolation and Culture of Rat Neocortical Neurons

The neocortical neurons utilized in this study were isolated from the embryos of specific pathogen-free (SPF) Sprague Dawley (SD) rats (Shanghai Lab. Animal Research Center, Shanghai, China) at embryonic day 16–18 (E16–E18). All animal procedures were approved by the Animal Ethics Committee of Southeast University, Jiangsu, China. All methods were performed in accordance with the relevant guidelines and regulations of this entity.

Before the experiment, multi-well plates intended for cell culture were coated with 0.1 mg/mL DPL (Gibco, Grand Island, NY, USA) for about 30 min. Then, the coating solution was aspirated, and the plates were washed with deionized water. The plates were air-dried in a laminar flow hood for later use. The female SPF SD rats were euthanized by CO_2_ asphyxiation followed by cervical dislocation. The neocortical tissue was isolated from embryos under low-temperature conditions and subsequently dissociated in TrypLE Express (Gibco, Grand Island, NY, USA). The culture medium used in the experiment consisted of Neurobasal Medium (Gibco, Grand Island, NY, USA) supplemented with 2% B-27 Supplement (Gibco, Grand Island, NY, USA), 0.5 mM GlutaMAX Supplement (Gibco, Grand Island, NY, USA), and 1% penicillin–streptomycin (BI, Kibbutz Beit Haemek, Israel). Neocortical neurons were dispersed in this medium and inoculated into the air-dried and coated plates. On the first day, the culture medium was fully replaced, and thereafter, half of the culture medium was replaced every 3–4 days. After a 14-day culture period, the neocortical neurons were used for the experiment.

### 4.2. Cell Viability Assay

The MTT assay was utilized to evaluate the viability of neurons in the experiment. First, after 14 days of culture, the neocortical neurons seeded in 96-well plates ($4\;\times \;10^{4}$ cells/well) were divided into a negative control group, a positive control group, and Ni^2+^ treatment groups. The culture medium of the negative control group was replaced with a normal culture medium, while that of the positive control group was replaced with a culture medium containing 0.7% acrylamide. The culture medium of the Ni^2+^ treatment groups was replaced with a culture medium containing 100, 200, 300, 400, 500, or 1000 μM NiCl_2_ (Sigma-Aldrich, St. Louis, MO, USA), respectively. After the neurons were further treated in the incubator for 24, 48, or 72 h, 200 μL of a normal culture medium containing 0.5 mg/mL MTT (BioFroxx, Einhausen, Germany) was added into all of the wells of the 96-well plates and was incubated in the incubator for another 2 h. Next, the culture medium was replaced with 150 μL of DMSO (Sinopharm, Shanghai, China) to dissolve the formed formazan crystals. To determine the optical density (OD) values of the solutions in each well, the wells were initially scanned using a spectrophotometer (Multiscan GO, Thermo-Scientific, Waltham, MA, USA) over a wavelength range of 300–700 nm. The results indicated that the OD values peaked at 553 nm. Subsequently, the OD values of each well were measured with 553 nm as the detection wavelength and 650 nm as the reference wavelength. The cell viability was defined as follows:

$$\mathrm{Viability}=\frac{OD_{553E}-OD_{650E}}{OD_{553\mathrm{NC}}-OD_{650\mathrm{NC}}}\times 100\%$$

where OD_553E_ and OD_650E_ represent the OD values measured at 553 nm and 650 nm, respectively, for the Ni^2+^ treatment groups; and OD_553NC_ and OD_650NC_ represent the OD values measured at the same wavelengths for the negative control group.

### 4.3. Measurement of the AIS’s Length

Immunofluorescence staining for the Ankyrin-G protein was used to identify and label the AIS. Neocortical neurons were seeded in 48-well plates at a density of $4\;\times {\;10}^{4}$ cells/well and cultured for 14 days. They were then divided into a control group and Ni^2+^ treatment groups. The culture medium of the control group was replaced with a normal culture medium, while that of the Ni^2+^ treatment groups was replaced with a culture medium containing 100 μM, 200 μM, or 500 μM NiCl_2_, respectively. After further treatment in the incubator for 48 h, the medium was removed and the neurons were washed with PBS. The neurons were first fixed with 4% paraformaldehyde for 20 min, followed by treatment with a Saponin-containing permeabilization solution (P0095, Beyotime, Shanghai, China) for 20 min to perforate the cell membrane. The neurons were then blocked with PBS containing 1% BSA (BioFroxx, Einhausen, Germany) at room temperature for 1 h. After removing the blocking solution, the primary antibody (Ankyrin-G Monoclonal Antibody, Invitrogen, Carlsbad, CA, USA) was added and incubated at room temperature for 1 h. Following three washes with PBS to remove the unbound primary antibody, the secondary antibody (Goat anti-Mouse IgG H&L Alexa Fluor 647, Abcam, Cambridge, UK) was added and incubated at room temperature for 1 h. Finally, immunofluorescence images of the AIS were captured using a high-content imaging system (ArrayScan XTI, ThermoFisher, Waltham, MA, USA). From the control group and the experimental groups treated with various concentrations of Ni^2+^, 20 images with clear staining were selected from each group. The lengths of the AISs were quantitatively measured using Image-Pro Plus 6.0 software.

### 4.4. Transcriptome Sequencing and Bioinformatics Analyses

Neurons seeded in 12-well plates ($5\times 10^{5}$ cells/well) were cultured for 14 days and then divided into a control group and Ni^2+^ treatment groups. The culture medium of the control group was replaced with a normal culture medium, while that of the Ni^2+^ treatment groups was replaced with a culture medium containing 100 μM, 200 μM, or 500 μM NiCl_2_, respectively. After further treatment in the incubator for 48 h, the culture medium was removed and the total RNA of neurons was then extracted from the control and Ni^2+^ treatment groups using Trizol (Invitrogen, Carlsbad, CA, USA).

Transcriptome sequencing and differential gene expression analysis were conducted by Beijing Biomarker Technology Co., Ltd. (Beijing, China). The purity and concentration of RNA were measured using a NanoDrop 2000 spectrophotometer (Thermo Scientific, Waltham, MA, USA), and RNA integrity was assessed using an Agilent 2100/LabChip GX system (Agilent, Santa Clara, CA, USA). Libraries were prepared from the RNA samples using a dedicated RNA sample preparation kit, followed by quality control. Sequencing was then performed on the NovaSeq 6000 platform (Illumina, San Diego, CA, USA). Differential expression analysis was conducted using edgeR. Genes with a *p*-value < 0.05 and $\left|\log_{2}\mathrm{FC}\right|\geq 1$ were identified as being DEGs.

The Metascape database was used to perform GO biological process and KEGG pathway enrichment analysis on the consistent DEGs in the Ni^2+^ treatment groups, with the results visualized using Origin 2025 software [30]. Additionally, a protein–protein interaction network was constructed using the String database, and the MCODE plugin in Cytoscape software, version 3.10.3 was employed to identify and extract key sub-networks [31,32,33].

### 4.5. ATP Content Measurement

The ATP content was measured in this study using an ATP detection kit (S0026, Beyotime, Shanghai, China). Neurons seeded in 48-well plates ($1.2\times 10^{5}$ cells/well) were cultured for 14 days and then divided into a control group and Ni^2+^ treatment groups. The culture medium of the control group was replaced with a normal culture medium, while that of the Ni^2+^ treatment groups was replaced with a culture medium containing 100 μM, 200 μM, or 500 μM NiCl_2_, respectively. After culturing the neurons in the incubator for an additional 48 h, the culture medium was removed, and the neurons were lysed using the lysis buffer provided in the kit. The supernatant was then collected. Subsequently, the ATP content (C_ATP_) and protein content (C_protein_) were determined, respectively, and the relative content of ATP was determined by the C_ATP_/C_protein_ ratio [63]. Finally, the ATP levels of each group of neurons were expressed as a percentage relative to the control group.

### 4.6. qRT-PCR

The same samples utilized for transcriptome sequencing were employed for qRT-PCR analysis. Four target genes—*Hk2*, *Ldha*, *Cd9*, and *Nfasc*—were selected for qRT-PCR validation. *Gapdh* was used as the internal reference gene. The PCR services were provided by Beijing Biomarker Technology Co., Ltd. (Beijing, China), and the primer sequences are listed in Table 1.

### 4.7. Statistical Analysis

Origin 2025 was employed for the statistical analysis of the experimental data. All statistical tests were two-tailed. Differences were considered statistically significant at *p* < 0.05.

## 5. Conclusions

This study demonstrates that the toxic effects of Ni^2+^ on rat neocortical neurons are closely associated with its concentration and the duration of exposure. Under low concentrations or short-term exposure, Ni^2+^ rapidly inhibits neuronal network excitability by blocking Ca^2+^ channels. Under high concentrations or prolonged exposure, Ni^2+^ not only downregulates the expression of *Cd9* and *Nfasc* genes but also activates the HIF-1α pathway, leading to enhanced glycolysis and elevated intracellular Ca^2+^ levels. These changes ultimately result in axonal structural damage and decreased cell viability.

## Figures and Tables

**Table 1:** The primer sequences for qRT-PCR.

Gene Symbol	Primers	Sequence (5′ to 3′)	TM (°C)
*Hk2*	*Hk2*-F	CCGTAGTGGACAAGATAAGAG	62
	*Hk2*-R	TGGCAAAGTGAGGATGAAG	
*Ldha*	*Ldha*-F	GCCGAGAGCATAATGAAGAA	62
	*Ldha*-R	CGTCAGGAGTCAGTGTCA	
*Cd9*	*Cd9*-F	ATATGGCGTTGAACTGCTGTG	62
	*Cd9*-R	GATGTGGAACTTGCTGTGGAA	
*Nfasc*	*Nfasc*-F	TGCTTATCGTCTGCTTCAT	62
	*Nfasc*-R	CTTGTTGTCCTCGTCACT	
*Gapdh* (internal reference)	*Gapdh*-F	GCCATCACTGCCACTCAGAAGAC	62
	*Gapdh*-R	ATGACCTTGCCCACAGCCTTG	

## Data Availability

The data presented in this study are available within the article and its Supplementary Materials. Additional information and data are available upon request from the corresponding author.

## Supplementary Materials

The following supporting information can be downloaded at: https://www.mdpi.com/article/10.3390/ijms26094014/s1.
