# Supplementary material for: Investigation of the Neurotoxicity Mechanisms of Ni2+ in Rat Neocortical Neurons Through Transcriptome Analysis
Source: Int J Mol Sci. 2025 Apr 24;26(9):4014. doi: 10.3390/ijms26094014 (PMC12072053; doi:10.3390/ijms26094014)
Supplement: Supplementary file 1 [file ijms-26-04014-s001.zip › ijms-3533748-supplementary.pdf]

Supplementary Materials

Investigation of the Neurotoxicity Mechanisms of Ni^{2+} in Rat Neocortical Neurons through Transcriptome Analysis

Chen Meng, Yang Lu, Yan Huang, Xiaoying Lü*

State Key Laboratory of Digital Medical Engineering,

Southeast University, Nanjing 210096, China

Index

Table S1. The viability of neurons after 24 h treatment of different concentrations of Ni^{2+} (n=6)...3

Table S2. The viability of neurons after 48 h treatment of different concentrations of Ni^{2+} (n=6)...3

Table S3. The viability of neurons after 72 h treatment of different concentrations of Ni^{2+} (n=6)...3

Table S4. The length (in pixel) of AIS after 48 h treatment of different concentrations of Ni^{2+}. 4

Table S5. Descriptive statistics of the data in Table S4. 22

Table S6. Up regulated genes in rat neocortical neurons after the treatment of 100, 200 or 500 μM Ni^{2+} for 48 h (n=3). 23

Table S7. Down regulated genes in rat neocortical neurons after the treatment of 100, 200 or 500 μM Ni^{2+} for 48 h (n=3). 25

Table S8. The result of GO biological processes enrichment. 30

Table S9. The result of KEGG enrichment. 39

Table S10. The ATP content of neurons after 48 h treatment of different concentrations of Ni^{2+} (n=4). 39

Table S11. The relative gene expression of *Hk2*, *Ldha*, *Cd9* and *Nfasc* after 48 h treatment of different concentrations of Ni^{2+} (n=3). 40

Table S12. A comparison between the effects of Ni^{2+} on gene expression in L929 cells and neocortical neurons. 42

Table S1. The viability of neurons after 24 h of treatment with different concentrations of Ni^{2+} (n=6).

Ni^{2+} (μM)	0	100	200	300	400	500	1000
Viability (%)	96.05	108.33	104.26	86.28	87.11	77.56	44.41
	94.45	116.54	92.90	92.38	84.81	75.26	44.23
	105.55	110.08	97.19	89.59	81.75	73.60	53.78
	99.59	116.09	99.12	99.20	87.21	79.83	50.97
	108.51	113.51	94.87	87.23	78.19	68.37	50.91
	95.85	95.59	87.03	81.42	74.69	74.84	45.43
Mean	100.00	110.02	95.89	89.35	82.29	74.91	48.29
Std.	5.78	7.78	5.84	6.05	5.07	3.89	4.09

Table S2. The viability of neurons after 48 h of treatment with different concentrations of Ni^{2+} (n=6).

Ni^{2+} (μM)	0	100	200	300	400	500	1000
Viability (%)	100.60	89.99	64.70	43.57	19.00	11.98	6.10
	99.20	106.39	66.69	47.07	18.84	12.97	6.11
	107.76	108.45	84.13	50.40	19.05	13.27	6.41
	105.18	99.67	73.33	49.63	18.18	13.56	7.14
	98.14	106.53	82.31	52.39	20.70	16.26	6.25
	89.12	90.32	68.63	43.38	18.13	14.65	5.99
Mean	100.00	100.22	73.30	47.74	18.98	13.78	6.33
Std.	6.48	8.35	8.22	3.72	0.93	1.49	0.42

Table S3. The viability of neurons after 72 h of treatment with different concentrations of Ni^{2+} (n=6).

Ni^{2+} (μM)	0	100	200	300	400	500	1000
Viability (%)	94.24	82.91	49.36	31.57	12.39	8.73	7.85
	86.34	99.13	60.32	41.01	15.14	8.35	7.82
	97.87	83.13	71.07	32.05	15.92	9.46	8.15
	103.27	113.14	50.60	24.20	12.09	11.51	7.90
	126.57	96.03	56.08	43.26	14.54	9.46	8.25
	91.71	85.23	60.27	39.17	13.68	8.08	8.28
Mean	100.00	93.26	57.95	35.21	13.96	9.27	8.04
Std.	14.21	11.93	7.93	7.19	1.53	1.24	0.21

Table S4. The length (in pixels) of AIS after 48 h of treatment with different concentrations of Ni^{2+}.

Ni^{2+} (μM)	0	100	200	500
	99.50	15.66	37.38	24.90
	29.97	26.38	39.87	23.66
	23.90	41.53	40.66	56.56
	48.14	11.83	64.73	42.73
	46.04	16.31	17.66	64.73
	48.04	95.50	9.66	30.14
	66.28	70.33	56.56	41.24
	38.31	31.21	65.53	32.87
	61.07	22.31	36.31	33.73
	21.14	49.80	68.67	38.49
	23.07	63.41	23.97	17.66
	44.07	35.97	43.11	17.24
	45.49	21.73	27.14	45.63
	72.60	75.70	47.46	42.11
	21.14	51.62	31.56	26.73
	53.04	57.94	43.14	37.31
	79.15	67.04	50.90	41.87
	164.41	72.97	32.24	30.97
	43.70	35.38	66.63	35.28
	50.21	54.94	29.66	66.91
	68.28	51.73	52.87	23.90
	18.49	26.73	36.31	25.66
	26.49	14.14	68.28	41.80
	52.53	52.97	31.56	43.53
	41.80	82.33	44.11	47.46
	29.24	71.43	54.60	43.63
	70.80	52.63	31.56	42.49
	12.83	13.83	40.87	41.70
	11.07	49.80	34.63	43.83
	110.30	51.21	24.24	32.49
	68.72	48.90	26.73	24.97
	117.38	80.08	38.80	55.38
	56.43	48.56	23.38	30.97
	57.11	23.90	26.97	19.31
	111.25	21.56	35.41	56.87
	93.57	17.07	22.24	51.94
	107.46	27.24	50.04	29.90
	76.53	78.67	37.24	18.90
	75.73	76.63	6.41	17.49
	94.05	78.60	20.31	26.21
	60.73	21.56	51.31	42.90

85.21	24.14	77.99	35.66
84.67	56.11	66.70	70.18
53.80	56.90	50.97	60.18
57.73	12.07	18.41	17.41
61.87	66.73	46.49	66.77
85.23	64.63	40.80	35.80
80.36	65.60	41.07	21.14
10.24	71.38	44.07	39.80
41.94	42.56	67.53	26.24
49.43	51.04	37.28	44.46
91.43	68.67	23.90	30.90
31.97	38.56	14.24	35.14
59.60	21.73	46.73	44.97
8.83	56.43	43.53	54.04
50.31	52.11	25.73	36.90
68.43	45.94	28.38	34.90
73.33	41.56	81.11	42.63
113.08	58.63	52.60	52.14
59.46	21.07	54.21	23.14
86.43	27.31	23.31	23.41
98.11	37.53	15.68	28.38
47.11	77.88	11.07	12.07
39.31	63.41	51.21	9.83
61.11	57.11	9.24	10.83
92.70	53.70	54.21	20.56
54.07	19.07	55.70	20.90
60.18	60.63	18.07	26.90
126.84	55.46	36.49	43.24
39.31	20.49	21.66	26.21
97.60	82.04	16.90	13.83
63.73	59.31	42.46	29.24
54.77	30.90	43.46	53.38
44.14	29.66	7.83	54.97
35.73	50.07	26.24	7.66
25.90	65.11	30.56	31.80
58.46	45.11	18.90	13.07
49.46	56.18	48.83	45.97
94.11	18.73	37.80	27.07
68.46	59.28	59.97	27.66
49.66	21.14	35.31	8.41
52.01	27.31	48.11	11.83
72.31	31.49	35.56	31.07
51.14	58.28	36.38	16.24
46.46	22.83	55.84	72.40

63.50	47.80	65.63	27.56
63.60	61.63	30.80	18.49
18.66	78.94	29.49	24.21
51.84	68.50	20.73	47.31
47.53	70.11	66.70	17.66
75.33	89.81	31.63	17.66
26.56	21.90	58.97	62.50
63.36	44.21	30.73	34.63
50.36	85.08	61.97	42.63
25.63	57.60	37.07	29.49
20.07	45.94	33.56	26.38
66.43	47.87	34.80	42.80
43.24	24.49	39.38	13.24
41.04	17.07	26.49	40.04
15.24	52.73	22.38	29.97
48.46	90.21	34.14	23.31
61.36	20.73	24.90	40.31
55.11	55.14	39.28	51.38
33.66	49.87	45.21	25.66
80.14	38.38	33.87	31.56
47.60	87.11	20.66	31.31
54.11	71.57	28.07	10.83
30.63	32.38	29.38	54.73
46.77	48.70	34.11	9.66
67.46	38.63	36.70	57.21
42.07	34.90	40.97	43.63
37.80	81.66	18.83	45.70
23.83	42.28	36.21	39.14
58.90	28.80	27.90	52.04
75.84	29.07	54.11	18.24
83.57	46.94	10.66	50.38
71.46	36.87	38.31	50.36
72.46	48.94	37.90	24.56
66.18	53.53	75.63	34.38
120.84	80.25	56.80	56.18
87.70	77.87	33.49	17.24
19.56	42.21	32.87	26.90
90.50	51.69	54.01	39.63
26.38	51.80	15.66	53.38
18.66	85.77	60.63	46.46
67.08	51.77	39.56	44.80
76.84	58.80	42.31	29.80
65.80	31.07	64.36	24.38
50.38	28.97	22.90	53.14

65.46	82.80	60.11	45.80
74.11	40.90	57.73	38.07
60.84	64.97	54.77	36.56
65.77	42.24	27.46	38.14
70.94	72.25	55.04	32.97
69.91	66.36	27.66	34.14
66.87	22.24	59.63	31.49
67.28	28.24	54.70	38.80
82.18	50.84	36.04	45.63
70.21	45.66	40.14	45.90
71.01	63.67	41.73	28.83
26.49	43.38	30.56	50.07
103.36	60.87	36.21	41.80
54.63	80.81	65.43	23.66
85.57	89.43	12.24	45.14
18.07	70.60	35.66	34.31
47.56	34.31	39.21	16.56
65.77	91.28	66.73	43.49
18.49	34.14	45.90	36.49
39.73	76.67	50.73	44.66
36.31	22.49	11.07	60.36
39.38	35.80	20.90	35.38
74.94	42.28	20.24	24.97
67.53	69.25	44.46	30.97
36.14	92.25	33.31	31.14
84.33	97.94	51.56	32.07
85.70	22.49	51.28	42.31
91.94	88.63	36.38	12.07
42.21	42.80	35.28	53.04
109.94	67.67	34.07	17.49
28.63	55.63	20.24	47.87
85.84	15.31	45.28	10.24
99.77	44.66	44.07	30.31
85.08	72.25	32.31	49.28
79.36	20.90	57.53	27.31
21.14	86.74	51.21	56.94
91.18	86.18	70.21	22.73
32.49	51.21	40.21	41.56
59.84	56.43	75.67	47.87
46.56	47.36	48.63	32.14
38.97	93.37	47.38	45.87
83.40	30.14	33.46	42.73
83.80	67.74	47.63	47.38
29.56	50.94	32.07	49.70

77.01	49.80	34.21	48.24
15.90	45.38	56.46	28.49
45.87	49.01	22.90	51.01
36.73	57.28	24.07	50.42
38.90	60.36	38.38	33.38
41.56	47.70	41.49	12.66
36.28	14.49	40.04	55.24
102.33	41.31	32.90	30.14
61.97	24.07	12.24	49.80
88.87	22.14	46.87	44.31
43.80	57.25	36.49	48.97
63.97	64.01	59.11	37.70
49.46	26.31	64.18	51.31
45.73	61.36	52.70	49.97
31.80	24.56	51.87	38.56
22.14	42.78	34.24	35.97
19.00	50.63	50.94	37.14
75.60	82.84	47.24	19.31
77.53	51.97	42.56	47.87
18.90	25.49	34.07	32.90
6.00	37.38	49.04	50.94
73.53	59.49	48.80	37.04
33.73	18.83	54.38	60.36
22.14	63.11	56.77	37.90
45.14	26.90	77.63	67.70
16.31	32.56	8.41	11.41
58.87	41.41	44.11	67.56
44.21	63.01	27.56	40.63
34.28	80.43	38.21	9.41
22.14	37.46	36.70	24.38
24.56	52.49	54.56	58.01
86.18	19.00	49.53	46.97
34.21	22.14	41.24	35.63
55.73	50.07	57.70	72.87
54.77	52.53	50.01	48.73
22.49	51.70	36.90	48.14
35.21	76.18	43.49	48.24
48.49	60.11	67.87	46.49
77.21	68.15	66.18	22.24
67.84	63.94	52.53	24.66
46.97	58.08	24.24	26.31
7.66	59.18	16.90	32.14
46.21	72.87	12.90	30.97
81.98	118.94	98.43	19.83

17.41	80.28	74.18	43.70
18.73	14.41	64.77	37.11
10.66	12.24	44.04	49.70
20.80	65.80	56.70	37.38
94.04	86.67	95.08	14.41
24.14	68.50	52.04	17.24
30.24	103.67	30.49	44.97
113.23	35.07	40.14	17.31
57.73	53.97	37.31	43.36
134.51	132.10	60.56	37.87
46.80	73.94	58.56	62.60
91.38	48.21	27.73	49.14
33.63	83.25	73.56	25.49
34.90	43.90	33.73	54.01
68.67	69.43	82.67	56.90
28.41	23.14	59.80	30.90
114.81	20.49	46.53	35.97
54.38	26.14	34.56	40.04
63.53	50.97	57.46	26.56
59.28	116.88	28.66	46.31
83.87	36.43	54.25	52.11
43.87	81.49	51.18	51.80
99.98	36.28	29.90	46.46
80.84	37.04	44.53	44.90
42.63	28.73	47.28	45.28
69.90	37.31	61.67	48.80
63.56	35.49	71.98	38.97
86.46	70.97	63.97	11.41
84.56	45.60	84.53	17.07
89.01	29.21	40.38	33.07
27.56	91.43	29.90	48.97
23.38	57.18	85.53	29.50
37.14	75.77	63.04	47.14
31.49	54.90	53.01	56.28
99.88	51.80	40.14	19.56
68.94	27.56	41.73	12.49
19.31	55.28	77.50	12.31
17.24	59.80	28.90	47.14
75.97	66.63	26.90	39.97
64.97	42.63	46.28	58.28
63.63	40.66	70.87	48.11
82.87	29.31	57.18	33.31
88.56	79.08	43.24	18.97
79.87	53.28	96.33	22.90

58.63	44.14	48.11	47.38
77.97	50.63	52.31	23.41
103.40	67.80	29.07	51.80
23.73	46.31	64.97	36.04
82.28	69.73	51.33	66.77
71.84	31.56	50.70	19.83
35.24	64.41	27.80	30.83
46.77	37.49	40.11	69.53
53.63	44.28	38.28	61.50
68.60	47.31	34.63	53.28
37.66	43.21	58.28	75.01
111.25	74.15	61.38	46.11
117.25	52.31	54.56	34.21
82.60	27.14	55.56	48.21
33.63	42.90	66.56	43.21
81.50	54.53	21.31	18.66
87.91	15.49	27.97	50.90
110.43	41.80	41.80	19.31
73.87	93.88	50.80	47.94
90.87	40.31	23.07	40.63
40.04	64.46	21.56	39.87
47.46	39.56	19.24	45.63
110.64	59.04	48.36	33.73
39.73	55.53	33.83	42.11
106.50	60.01	27.66	44.21
46.46	69.11	65.38	45.36
35.46	78.74	45.21	14.24
63.77	67.77	51.70	33.90
17.66	67.21	12.66	26.49
11.07	118.15	63.04	49.70
17.41	32.87	49.14	18.66
17.66	52.87	44.38	43.21
31.38	62.87	74.11	32.87
23.97	25.31	58.49	18.14
91.08	8.66	46.04	17.24
23.24	13.49	48.80	46.73
108.84	76.84	50.04	47.04
68.91	45.46	43.66	14.24
62.50	22.31	44.94	42.31
55.97	58.53	26.07	73.28
39.90	56.38	39.38	26.21
79.36	51.38	51.01	30.31
48.46	57.60	24.31	9.24
47.80	20.24	23.38	32.38

50.28	57.28	56.11	20.90
42.56	58.87	48.97	23.66
56.80	84.18	37.63	35.90
70.97	55.87	49.53	26.97
25.49	49.56	39.36	44.73
28.90	8.41	58.11	19.56
30.83	12.24	58.04	33.63
78.43	13.66	20.07	39.38
77.63	85.43	34.38	37.97
46.63	20.73	44.87	43.11
34.87	53.80	49.14	54.31
55.73	58.77	42.63	48.80
45.94	55.94	63.87	41.38
93.12	94.21	38.97	37.87
57.18	33.41	29.90	32.66
10.24	42.49	43.73	13.07
89.56	87.11	41.11	30.43
136.81	51.46	37.38	60.53
58.97	86.21	34.24	54.53
77.28	96.91	39.56	40.97
44.87	93.71	73.31	44.97
51.28	93.64	50.87	36.31
117.64	64.38	48.73	32.87
100.81	39.94	55.73	49.77
57.97	26.80	34.38	45.97
89.25	73.40	19.97	18.24
27.49	64.28	17.07	64.21
30.56	59.01	40.63	43.56
42.21	52.97	32.49	50.77
128.81	124.47	29.46	12.73
58.21	78.46	33.49	19.90
102.05	68.98	44.73	75.80
27.21	77.15	46.80	43.63
14.07	18.66	32.90	50.21
81.15	117.88	44.04	41.28
48.38	75.05	50.94	33.28
81.33	58.90	53.83	42.00
92.18	35.46	42.63	22.14
29.97	50.11	40.46	61.14
41.80	52.31	37.73	42.04
42.63	58.36	27.46	44.73
25.14	37.38	34.38	20.07
112.04	55.94	45.01	49.78
66.46	50.04	44.38	33.21

57.28	30.90	24.80	31.56
58.25	55.14	42.56	10.66
110.36	56.70	48.90	34.73
43.90	72.11	45.11	27.14
52.90	46.97	16.66	32.80
9.66	36.21	24.49	43.46
87.25	40.63	39.97	34.24
76.21	74.14	21.41	25.90
84.74	33.46	38.04	27.73
33.73	69.11	65.11	66.25
64.04	75.70	83.18	69.23
78.70	12.24	32.73	44.04
66.28	56.21	33.21	47.07
40.41	59.53	38.49	52.93
32.80	67.25	27.24	39.63
49.38	47.90	57.70	42.73
75.53	35.28	74.11	38.87
87.18	60.94	14.14	24.56
44.07	83.84	17.07	37.07
110.95	63.77	56.70	34.04
15.49	66.28	15.41	55.46
62.08	37.11	39.21	57.36
34.63	71.46	42.63	37.80
24.66	44.36	38.66	45.56
52.53	33.14	12.41	36.87
125.23	84.36	22.14	37.38
61.38	28.07	29.49	17.14
78.21	26.07	24.24	15.00
56.43	36.90	20.49	39.97
12.24	19.31	40.56	41.46
38.38	14.49	52.28	43.14
22.38	21.49	41.87	27.73
42.04	44.94	47.87	39.74
9.24	34.04	52.83	49.97
11.07	9.00	20.07	44.87
96.23	60.63	7.24	45.04
66.63	27.14	20.83	38.73
47.77	23.56	23.73	45.21
54.04	76.97	50.38	37.97
83.43	38.38	30.90	15.66
62.63	62.04	61.77	28.49
34.14	64.94	39.28	32.97
94.33	63.67	48.11	17.41
59.53	23.56	31.70	34.31

127.92	28.31	38.83	32.66
102.63	79.36	46.97	39.63
54.28	44.31	13.83	44.14
43.97	80.80	31.21	16.41
50.53	19.14	53.97	34.04
31.46	9.66	47.07	26.56
41.73	44.56	53.80	28.07
63.21	28.21	52.04	20.49
77.67	62.14	30.14	31.14
25.90	31.38	60.56	10.83
104.20	57.04	52.53	29.90
39.21	49.70	9.83	31.07
20.24	51.56	11.41	42.70
53.94	32.46	6.83	14.24
79.67	73.63	57.77	28.66
55.38	15.83	56.53	29.97
43.46	11.83	28.04	32.73
81.70	58.70	40.53	47.04
40.53	80.28	69.28	55.46
47.43	61.46	44.97	45.90
33.38	21.56	65.63	14.41
44.87	30.14	11.41	28.14
60.28	49.97	19.66	28.66
52.44	34.83	48.94	54.67
77.11	46.66	60.60	23.49
15.31	15.90	81.33	27.38
28.56	48.31	93.23	33.63
98.61	53.21	56.04	44.97
34.46	78.77	34.90	18.66
34.04	46.36	49.36	16.49
20.49	36.97	36.07	35.38
62.63	42.31	55.14	23.73
30.56	59.36	52.87	32.56
62.77	55.94	29.07	17.00
66.43	80.23	21.56	24.38
45.73	44.80	50.97	31.80
89.84	38.11	25.31	13.73
45.87	48.04	44.31	9.83
70.04	58.21	48.87	26.90
55.04	10.66	36.90	22.56
72.11	58.60	39.28	27.81
54.36	51.70	61.60	31.56
76.98	44.56	27.73	56.53
43.53	113.47	67.18	20.83

59.11	30.24	59.14	23.66
43.46	73.84	53.56	40.63
47.14	108.54	46.63	43.56
41.78	66.01	36.87	50.14
40.87	58.80	13.49	27.41
59.87	68.01	49.21	38.87
60.28	70.87	47.04	19.24
74.19	44.63	41.70	33.41
82.94	27.56	59.94	45.87
81.98	63.21	61.36	33.66
57.28	7.66	35.49	25.73
62.60	33.14	40.11	32.56
38.80	26.80	35.31	46.28
46.73	38.80	23.31	32.66
67.53	96.67	50.36	23.24
32.49	68.94	40.38	20.66
43.46	36.87	19.90	42.90
17.83	45.63	55.38	33.41
60.73	12.24	60.04	65.90
53.90	21.66	44.73	16.66
89.57	41.04	39.90	21.66
58.87	45.80	35.04	24.24
34.97	79.60	36.73	51.14
55.53	35.21	41.04	37.04
49.73	15.83	36.90	45.14
83.18	76.60	32.97	35.63
36.87	47.28	41.80	57.43
36.31	41.21	47.83	52.21
68.08	85.25	60.36	42.25
54.84	34.24	64.01	32.49
37.31	9.66	12.00	24.83
93.36	42.80	6.83	34.73
45.60	80.46	48.90	30.38
69.84	37.70	65.77	46.28
52.80	75.63	38.80	42.04
58.60	86.84	30.66	54.80
34.21	69.74	43.21	27.73
15.07	21.00	9.83	39.56
49.73	56.87	53.11	22.56
44.28	22.73	54.36	47.21
59.91	45.70	30.49	35.97
66.84	71.25	27.38	45.21
36.63	78.77	44.11	49.53
58.36	51.56	50.28	37.97

44.24	89.36	5.83	46.56
30.56	124.91	15.66	58.14
111.12	22.38	33.90	25.14
99.28	40.04	24.73	39.14
59.31	28.80	43.90	32.38
60.87	67.46	21.83	29.56
68.94	67.63	32.56	26.07
99.67	47.70	29.31	42.87
54.38	52.77	43.56	40.49
56.94	76.87	45.56	48.80
70.80	34.24	49.63	46.63
52.86	40.70	58.25	65.11
46.21	51.63	34.49	43.73
30.80	60.73	49.73	36.46
50.60	56.46	47.14	51.21
49.90	22.83	54.04	53.66
13.83	17.31	19.66	48.87
50.94	50.11	104.87	63.56
57.73	12.24	37.21	42.70
80.63	9.83	46.07	54.08
73.08	35.04	48.73	69.04
50.63	38.46	36.21	51.07
44.21	11.07	49.21	29.73
83.01	61.28	38.41	31.97
70.77	23.95	33.56	58.87
51.21	43.53	56.56	22.31
49.70	30.66	41.83	63.46
55.28	34.14	42.31	24.66
55.70	36.56	33.97	38.28
27.80	42.80	68.53	54.28
22.97	66.53	30.38	50.87
38.38	38.73	47.90	21.97
24.63	63.25	30.14	42.21
39.87	33.21	55.49	40.38
48.56	54.11	52.63	43.49
42.63	35.97	38.49	51.04
57.08	54.36	30.31	42.56
63.87	52.53	67.53	41.24
40.90	76.04	19.35	50.21
46.36	19.24	57.53	41.97
79.36	53.63	55.97	41.04
38.24	48.14	36.66	43.63
21.31	70.25	32.56	40.90
53.36	68.01	57.63	44.21

48.38	31.24	20.66	21.31
85.01	13.66	14.41	37.56
68.80	13.24	51.70	21.66
66.18	50.94	45.21	11.41
113.87	32.31	67.91	48.53
39.14	24.73	45.04	17.07
97.53	45.97	41.31	42.90
39.14	93.18	48.31	39.31
40.46	71.67	93.88	48.63
61.97	48.04	42.04	31.63
60.94	78.84	46.49	39.97
86.25	49.73	30.38	46.97
70.80	46.53	46.68	37.38
51.53	109.15	48.36	41.63
38.80	94.70	19.41	38.07
22.07	84.05	24.14	47.38
55.63	76.77	36.70	46.73
68.43	50.77	43.90	31.56
90.67	48.56	35.87	39.66
58.51	39.31	64.21	28.24
99.23	22.31	39.07	25.31
22.83	37.66	39.49	12.24
45.18	64.36	48.21	11.24
50.56	53.56	37.38	
24.66	55.24	25.24	
39.80	40.63	10.07	
72.18	25.24	20.49	
46.80	37.49	48.53	
42.77	34.28	45.31	
48.90	63.73	67.60	
61.91	53.43	11.24	
83.38	49.94	64.04	
86.88	48.81	50.14	
50.60	87.97	46.56	
84.67	69.63	32.04	
87.98	41.31	48.80	
49.56	54.53	61.84	
36.66	55.63	57.84	
50.14	58.70	35.66	
58.14	42.53	17.41	
70.08	26.97	47.77	
47.56	52.87	42.80	
47.18	73.97	22.90	
99.46	37.46	30.38	

58.56	33.04	41.56
57.18	57.73	24.24
71.80	40.38	30.97
61.94	60.73	29.31
46.46	93.30	25.49
33.63	61.43	19.41
36.21	53.07	32.73
45.38	59.04	23.14
71.56	44.80	38.46
70.11	71.11	39.73
75.63	91.47	20.07
44.80	20.49	52.53
75.08	51.36	23.14
20.90	48.28	39.04
32.66	71.08	19.07
51.56	54.73	48.31
78.80	44.66	31.66
53.14	45.38	36.38
38.14	25.97	41.56
41.14	34.87	23.24
65.18	59.04	37.90
102.91	67.18	48.11
49.28	75.14	55.87
82.08	17.07	51.97
67.07	52.53	47.33
64.01	61.46	20.90
57.01	44.53	62.94
72.25	33.70	44.04
35.46	56.24	55.56
17.73	46.38	88.87
76.74	42.53	22.66
57.60	81.46	62.28
46.28	41.49	24.14
70.84	73.53	64.87
40.00	55.11	56.28
56.43	55.70	41.63
24.73	55.70	34.49
67.46	75.87	54.77
51.38	67.70	47.97
67.36	26.80	30.80
23.63	43.80	57.38
26.38	11.49	44.56
61.70	51.53	41.94
41.94	17.66	39.80

77.18	45.53	52.28
63.77	56.49	27.80
53.28	54.67	22.07
66.49	46.70	46.56
55.60	69.18	8.24
84.67	46.11	41.73
59.70	28.97	40.80
63.73	55.67	48.24
43.11	48.71	45.80
29.38	60.93	13.83
43.97	21.43	45.07
39.97	66.91	37.46
62.98	56.31	68.18
49.04	61.05	11.41
21.31	51.17	41.36
100.64	64.02	42.04
85.60	34.87	31.90
82.40	57.99	77.20
31.21	40.24	56.73
49.80	42.85	33.73
36.14	42.05	48.28
21.49	62.82	32.49
42.07	57.90	15.41
47.46	67.63	65.67
28.21	65.72	62.97
86.38	35.83	30.38
65.36	33.32	46.04
38.97	23.63	28.49
45.97	56.29	32.49
64.07	51.74	50.36
76.77	61.28	30.56
52.04	41.43	75.04
45.31	56.21	77.08
67.43	38.45	26.31
83.21	37.19	60.04
64.87	67.10	23.07
126.30	65.50	36.70
79.84	56.90	64.11
53.97	66.46	48.97
29.80	35.32	41.38
50.25	19.92	44.46
70.01	48.37	51.80
71.21	18.38	33.56
50.38	46.65	41.07

51.01	49.91	47.80
45.94	45.52	52.77
16.56	57.15	54.21
22.90	31.07	42.21
20.31	31.97	29.49
40.31	46.87	33.24
39.63	58.63	66.25
48.31	33.96	51.46
49.63	33.53	38.97
80.46	69.58	92.74
28.38	23.88	36.97
57.66	48.00	79.80
51.70	12.73	78.31
100.08	30.24	51.21
62.31	27.75	70.63
35.70	72.17	51.63
58.36	41.27	25.24
73.18	55.66	36.63
75.01	91.96	61.70
35.90	55.21	27.31
33.00	42.51	61.91
55.46	30.47	55.04
43.66	67.47	45.31
63.56	33.00	43.46
37.73	46.83	38.31
78.60	55.72	24.73
80.36	51.26	26.14
34.56	81.58	38.63
31.04	54.26	25.07
32.66	47.90	47.04
85.08	48.28	72.28
55.38	40.45	39.63
59.94	31.40	29.63
26.31	45.18	22.73
59.91	39.69	22.24
30.80	53.26	23.90
48.77	23.96	13.90
29.38	61.19	50.46
36.14	55.02	46.97
54.11	13.06	30.56
68.60	47.12	41.97
58.31	39.62	47.97
40.56	72.15	49.94
12.49	34.92	41.73

59.28	51.20	60.84
44.97	58.61	43.80
64.87	35.52	53.63
64.07	54.62	38.14
50.49	60.73	33.21
64.11	64.42	60.36
48.97	48.47	71.77
29.80	35.80	77.58
78.53	46.31	35.46
83.25	50.97	15.31
24.97	13.29	38.87
22.31	35.31	79.43
9.66	21.49	34.66
55.28	33.80	56.11
83.43	40.80	35.21
72.53	72.53	61.01
39.14	55.94	33.97
35.73	43.46	40.73
79.71	69.43	45.46
44.97	30.38	52.46
47.31	114.10	38.14
56.84	71.50	77.58
22.38	72.36	64.97
59.63	50.84	32.31
69.04	70.43	32.56
31.21	52.97	29.80
48.21	52.56	41.73
30.21	67.08	36.73
25.14	82.28	18.90
60.18	43.56	17.07
83.84	23.49	54.28
83.60	17.49	48.28
56.36	46.31	46.87
74.01	31.38	34.38
29.31	22.14	74.46
57.38	12.83	27.80
26.73	65.28	58.67
14.41	66.46	12.07
41.97	57.77	15.31
68.31	78.77	19.00
48.01	45.14	55.49
51.14	48.90	30.97
71.08	33.38	44.73
102.01	38.97	54.80

59.84	46.28	34.56
56.11	10.90	49.46
78.28	32.73	48.49
67.43	89.53	50.31
27.21	30.90	48.87
77.80	57.56	27.24
71.57	39.14	42.49
32.90	66.60	72.91
30.97	32.56	30.21
79.46	25.49	51.11
51.36	37.14	51.18
95.08	99.91	18.14
54.87	68.77	50.21
56.77	51.77	59.56
54.43	73.60	56.59
43.66	86.70	49.46
56.60	54.80	58.80
63.66	59.63	39.80
47.46	42.56	46.14
83.43	22.90	49.63
51.14	33.63	46.94
59.21	44.14	26.60
21.07	64.84	58.97
63.60	69.31	26.56
49.46	46.21	32.63
49.07	12.49	44.21
85.25	41.53	30.80
70.36	86.77	50.38
100.01	60.08	36.97
79.53	71.97	31.07
40.49	28.39	14.66
52.36	33.31	39.38
50.87	46.73	60.56
54.87	36.14	28.66
69.91	79.21	48.53
49.73	38.28	32.07
43.53	60.43	74.01
68.91	65.11	37.28
62.28	44.80	36.56
	56.66	27.90
	42.21	25.80
	69.87	34.04
	44.56	24.97
	62.70	36.97

26.38	43.90
93.28	24.83
62.38	17.83
58.80	28.07
49.87	34.97
61.04	57.01
59.67	52.04
32.31	37.31
10.66	41.28
113.36	60.97
48.73	42.31
66.25	65.24
22.73	37.70
71.70	50.94
52.14	39.80
72.77	27.41
51.73	50.38
73.77	50.21
45.28	39.80
48.14	24.49
19.90	28.07
73.50	56.77
26.97	60.80
38.56	17.90
27.14	32.73
32.97	33.83
30.70	55.56
	27.56
	59.49
	31.49
	51.94
	30.97
	41.97
	51.80

Table S5. Descriptive statistics of the data in Table S4.

Ni^{2+} (μM)	n	Min	Q1	Median	Q3	Max
0	784	39.31	54.7	72.16	164.41	39.31
100	816	34.95	50.37	64.58	132.1	34.95
200	823	30.8	41.56	52.28	104.87	30.8
500	548	26.77	37.83	46.46	75.8	26.77

Table S6. Upregulated genes in rat neocortical neurons after the treatment with 100, 200, or 500 μM Ni^{2+} for 48 h (n=3).

Gene ID	100 μM Ni^{2+} (log_2FC)	200 μM Ni^{2+} (log_2FC)	500 μM Ni^{2+} (log_2FC)
ENSRNOG00000001766	2.46	2.87	2.84
ENSRNOG00000037251	1.10	1.52	1.15
ENSRNOG00000006378	2.33	2.50	1.02
ENSRNOG00000014320	2.23	4.08	2.05
ENSRNOG00000003950	1.67	2.29	1.92
ENSRNOG00000007839	1.14	2.10	1.75
ENSRNOG00000005206	1.03	2.44	1.73
ENSRNOG00000050669	1.03	1.26	1.56
ENSRNOG00000040279	1.54	1.26	1.13
ENSRNOG00000014975	1.24	1.62	1.39
ENSRNOG00000045738	1.28	1.76	2.46
ENSRNOG00000013009	1.40	2.22	3.04
ENSRNOG00000001585	1.16	2.16	1.12
ENSRNOG00000027276	1.35	2.39	1.18
ENSRNOG00000001214	1.41	1.84	2.13
ENSRNOG00000058593	1.11	1.09	1.06
ENSRNOG00000028335	1.21	2.23	1.72
ENSRNOG00000061862	1.38	1.80	1.11
ENSRNOG00000013390	1.49	1.74	1.09
ENSRNOG00000049104	1.42	1.69	1.09
ENSRNOG00000005569	1.35	2.06	3.86
ENSRNOG00000015075	1.07	1.38	2.20
ENSRNOG00000008423	1.17	2.55	1.83
ENSRNOG00000031031	2.86	2.86	1.17
ENSRNOG00000014859	1.09	2.36	1.29
ENSRNOG00000032307	1.69	2.64	1.42
ENSRNOG00000005159	1.39	2.75	2.33
ENSRNOG00000024479	1.61	3.18	1.10
ENSRNOG00000029956	2.15	2.20	1.06
ENSRNOG00000039057	1.04	1.53	1.22
ENSRNOG00000005578	1.33	1.33	1.09
ENSRNOG00000025587	1.15	1.44	1.01
ENSRNOG00000036913	1.91	2.18	1.02
ENSRNOG00000008765	2.33	3.37	1.82
ENSRNOG00000033942	1.16	1.99	1.28
ENSRNOG00000015290	1.18	1.28	1.74
ENSRNOG00000027030	2.11	2.31	2.81
ENSRNOG00000018690	1.11	1.55	1.03
ENSRNOG00000016298	1.81	1.85	1.02
ENSRNOG00000003732	1.24	2.46	2.11

ENSRNOG00000025704	1.37	1.60	1.16
ENSRNOG00000003675	1.51	2.77	1.64
ENSRNOG00000050476	1.51	1.37	1.57
ENSRNOG00000006116	1.81	2.57	4.90
ENSRNOG00000014089	1.51	1.41	1.13
ENSRNOG00000007706	1.70	2.19	1.11
ENSRNOG00000021053	1.25	2.09	3.16
ENSRNOG00000019598	2.60	2.69	2.46
ENSRNOG00000030034	1.08	1.95	1.50
ENSRNOG00000027921	1.88	2.11	1.42
ENSRNOG00000050655	1.61	1.86	3.10
ENSRNOG00000031335	2.79	3.91	5.58
ENSRNOG00000003581	1.25	1.37	1.93
ENSRNOG00000036664	1.31	1.63	2.46
ENSRNOG00000022711	1.82	2.94	1.85
ENSRNOG00000004918	1.03	2.00	2.02
ENSRNOG00000003756	1.38	1.84	1.41
ENSRNOG00000007814	1.78	2.06	2.79
ENSRNOG00000015383	1.22	2.20	1.25
ENSRNOG00000053366	1.87	2.13	1.07
ENSRNOG00000009735	1.11	1.92	1.40
ENSRNOG00000047499	1.26	1.44	3.20

Table S7. Downregulated genes in rat neocortical neurons after the treatment with 100, 200, or 500 μM Ni^{2+} for 48 h (n=3).

Gene ID	100 μM Ni^{2+} (log_2FC)	200 μM Ni^{2+} (log_2FC)	500 μM Ni^{2+} (log_2FC)
ENSRNOG00000013330	-1.23	-2.34	-3.44
ENSRNOG00000021750	-1.42	-1.90	-1.32
ENSRNOG00000019403	-1.70	-3.37	-1.98
ENSRNOG00000000566	-1.82	-1.61	-1.09
ENSRNOG00000007728	-1.25	-2.75	-2.36
ENSRNOG00000008915	-1.40	-2.63	-3.09
ENSRNOG00000018950	-1.75	-7.45	-9.03
ENSRNOG00000002365	-1.06	-2.16	-2.99
ENSRNOG00000049766	-2.04	-2.30	-1.81
ENSRNOG00000025948	-1.57	-7.81	-7.19
ENSRNOG00000003266	-1.20	-1.33	-1.62
ENSRNOG00000017748	-1.48	-6.76	-9.51
ENSRNOG00000024631	-1.58	-3.70	-3.07
ENSRNOG00000000456	-1.05	-3.12	-2.60
ENSRNOG00000002963	-1.18	-4.25	-7.71
ENSRNOG00000049983	-1.24	-1.27	-1.42
ENSRNOG00000003105	-1.87	-2.77	-2.85
ENSRNOG00000038365	-2.48	-3.29	-3.94
ENSRNOG00000055221	-1.69	-4.53	-4.76
ENSRNOG00000002425	-1.53	-2.07	-1.51
ENSRNOG00000003538	-1.07	-2.03	-1.79
ENSRNOG00000016512	-1.08	-1.45	-1.78
ENSRNOG00000021023	-1.00	-4.85	-5.31
ENSRNOG00000011145	-2.78	-1.72	-2.55
ENSRNOG00000031266	-1.06	-1.99	-2.04
ENSRNOG00000019179	-2.61	-2.50	-2.45
ENSRNOG00000015321	-1.28	-1.76	-2.78
ENSRNOG00000034191	-1.28	-5.01	-8.18
ENSRNOG00000049407	-1.25	-2.23	-1.97
ENSRNOG00000008592	-1.36	-2.74	-1.96
ENSRNOG00000008595	-1.03	-1.98	-1.82
ENSRNOG00000007905	-1.03	-2.57	-1.93
ENSRNOG00000024457	-1.23	-1.91	-4.70
ENSRNOG00000003069	-2.23	-3.55	-2.06
ENSRNOG00000007431	-1.60	-2.36	-1.50
ENSRNOG00000057542	-1.09	-6.70	-11.19
ENSRNOG00000008040	-1.31	-1.55	-3.33
ENSRNOG00000015406	-2.10	-7.02	-2.77
ENSRNOG00000053055	-1.91	-2.34	-1.89
ENSRNOG00000020081	-1.46	-2.28	-2.66

ENSRNOG00000017646	-1.22	-3.00	-4.26
ENSRNOG00000018358	-1.12	-2.83	-3.63
ENSRNOG00000019556	-1.72	-5.83	-3.53
ENSRNOG00000013215	-1.38	-2.30	-2.34
ENSRNOG00000010549	-2.10	-3.35	-3.62
ENSRNOG00000014659	-1.16	-1.51	-1.44
ENSRNOG00000016496	-1.35	-2.89	-3.09
ENSRNOG00000012972	-2.02	-2.31	-1.62
ENSRNOG00000021521	-1.50	-4.33	-3.74
ENSRNOG00000001159	-1.55	-3.79	-8.13
ENSRNOG00000007266	-2.33	-3.92	-3.46
ENSRNOG00000027739	-1.97	-3.50	-3.95
ENSRNOG00000028274	-1.02	-4.84	-4.94
ENSRNOG00000045670	-1.33	-1.92	-2.11
ENSRNOG00000010076	-2.29	-7.98	-3.60
ENSRNOG00000025378	-1.05	-2.09	-2.16
ENSRNOG00000009694	-3.14	-4.69	-6.58
ENSRNOG00000012748	-1.17	-1.83	-1.08
ENSRNOG00000015318	-3.10	-3.39	-3.50
ENSRNOG00000022932	-1.14	-1.12	-1.59
ENSRNOG00000008543	-2.65	-3.23	-1.62
ENSRNOG00000006623	-2.47	-2.57	-2.27
ENSRNOG00000050697	-2.08	-2.28	-1.14
ENSRNOG00000033261	-1.22	-1.43	-2.19
ENSRNOG00000010652	-1.67	-3.22	-2.55
ENSRNOG00000018874	-1.35	-2.07	-1.76
ENSRNOG00000011526	-1.03	-1.74	-3.31
ENSRNOG00000045558	-1.65	-1.68	-2.04
ENSRNOG00000014684	-1.06	-2.72	-2.29
ENSRNOG00000048914	-2.09	-2.81	-2.17
ENSRNOG00000029778	-2.12	-3.14	-4.49
ENSRNOG00000027229	-1.01	-1.57	-2.01
ENSRNOG00000017693	-1.03	-6.42	-6.92
ENSRNOG00000021323	-1.57	-1.74	-1.71
ENSRNOG00000005695	-2.34	-2.33	-5.37
ENSRNOG00000024825	-1.19	-2.58	-5.25
ENSRNOG00000015554	-1.49	-1.32	-1.15
ENSRNOG00000024712	-2.28	-3.07	-3.73
ENSRNOG00000016769	-1.35	-2.54	-3.94
ENSRNOG00000034134	-1.16	-6.17	-4.45
ENSRNOG00000008534	-1.02	-2.39	-2.40
ENSRNOG00000028092	-2.70	-2.38	-2.35
ENSRNOG00000005931	-2.02	-2.52	-2.94
ENSRNOG00000031126	-1.66	-1.00	-1.12

ENSRNOG00000011719	-1.78	-2.17	-1.13
ENSRNOG00000009884	-1.87	-3.37	-3.35
ENSRNOG00000003273	-3.03	-2.60	-5.08
ENSRNOG00000022852	-1.77	-5.54	-3.07
ENSRNOG00000002273	-1.77	-1.55	-1.30
ENSRNOG00000039390	-1.75	-1.55	-1.48
ENSRNOG00000007457	-1.39	-2.40	-1.87
ENSRNOG00000049714	-1.98	-3.10	-2.61
ENSRNOG00000016873	-2.73	-2.56	-2.02
ENSRNOG00000004448	-1.16	-1.66	-4.38
ENSRNOG00000019926	-2.71	-2.17	-1.47
ENSRNOG00000000658	-1.43	-2.81	-3.35
ENSRNOG00000012160	-1.52	-1.95	-1.70
ENSRNOG00000016342	-1.20	-1.70	-3.78
ENSRNOG00000012995	-2.12	-2.29	-2.16
ENSRNOG00000011971	-1.61	-1.33	-1.04
ENSRNOG00000021919	-1.21	-1.41	-2.99
ENSRNOG00000018239	-1.05	-1.75	-1.44
ENSRNOG00000011427	-1.33	-2.85	-2.40
ENSRNOG00000059956	-1.69	-2.64	-1.49
ENSRNOG00000051548	-2.61	-3.90	-4.93
ENSRNOG00000007062	-1.08	-1.51	-1.50
ENSRNOG00000011323	-1.68	-1.61	-1.02
ENSRNOG00000011320	-1.33	-2.80	-3.05
ENSRNOG00000010986	-3.65	-8.25	-9.14
ENSRNOG00000042041	-1.15	-6.16	-7.85
ENSRNOG00000000138	-1.09	-1.41	-1.75
ENSRNOG00000027456	-1.27	-1.96	-2.02
ENSRNOG00000059500	-1.00	-6.25	-5.73
ENSRNOG00000003893	-1.41	-3.62	-3.72
ENSRNOG00000038297	-1.08	-2.50	-3.24
ENSRNOG00000013954	-1.16	-2.22	-2.97
ENSRNOG00000050539	-1.25	-1.04	-3.49
ENSRNOG00000042620	-2.82	-3.24	-2.30
ENSRNOG00000002461	-1.96	-1.90	-2.14
ENSRNOG00000016558	-2.52	-6.64	-6.28
ENSRNOG00000007561	-1.61	-3.53	-4.00
ENSRNOG00000028384	-1.57	-2.43	-3.33
ENSRNOG00000017803	-1.19	-8.23	-3.17
ENSRNOG00000054331	-1.12	-1.63	-1.05
ENSRNOG00000028083	-1.28	-1.84	-1.24
ENSRNOG00000045838	-1.28	-1.98	-1.24
ENSRNOG00000022764	-1.27	-7.20	-7.00
ENSRNOG00000015774	-1.41	-3.47	-2.73

ENSRNOG00000001192	-1.09	-3.70	-3.37
ENSRNOG00000017605	-1.09	-1.62	-1.90
ENSRNOG00000049771	-1.21	-2.65	-2.39
ENSRNOG00000009751	-1.88	-2.40	-1.48
ENSRNOG00000019494	-1.05	-1.80	-1.18
ENSRNOG00000019496	-2.68	-3.19	-2.06
ENSRNOG00000058243	-1.51	-2.77	-3.36
ENSRNOG00000026604	-1.45	-2.25	-1.94
ENSRNOG00000033588	-1.98	-5.30	-6.20
ENSRNOG00000014936	-1.16	-1.74	-2.18
ENSRNOG00000008989	-1.23	-1.74	-1.86
ENSRNOG00000007367	-1.26	-1.77	-2.05
ENSRNOG00000029148	-1.04	-1.54	-3.42
ENSRNOG00000028238	-1.81	-3.89	-2.86
ENSRNOG00000008837	-1.46	-1.83	-2.36
ENSRNOG00000020653	-1.26	-1.98	-1.01
ENSRNOG00000010263	-2.91	-7.85	-11.74
ENSRNOG00000047000	-2.54	-2.20	-2.41
ENSRNOG00000003098	-1.85	-2.41	-2.13
ENSRNOG00000010716	-1.12	-3.15	-6.45
ENSRNOG00000006068	-1.08	-1.56	-1.49
ENSRNOG00000048273	-1.73	-7.02	-8.22
ENSRNOG00000039284	-1.33	-1.82	-1.61
ENSRNOG00000017496	-1.16	-4.70	-5.72
ENSRNOG00000003833	-1.80	-1.75	-1.07
ENSRNOG00000021400	-2.71	-2.45	-2.33
ENSRNOG00000004708	-1.37	-1.31	-1.32
ENSRNOG00000022567	-1.16	-4.22	-4.48
ENSRNOG00000052564	-1.96	-2.29	-2.52
ENSRNOG00000005669	-1.21	-2.81	-2.18
ENSRNOG00000025384	-1.01	-2.32	-2.85
ENSRNOG00000012216	-1.80	-2.49	-2.94
ENSRNOG00000001911	-1.74	-2.08	-1.81
ENSRNOG00000026324	-1.03	-2.07	-3.30
ENSRNOG00000019751	-1.39	-3.94	-4.54
ENSRNOG00000025676	-1.25	-4.76	-8.77
ENSRNOG00000043007	-1.71	-1.38	-1.14
ENSRNOG00000018369	-1.59	-3.68	-4.16
ENSRNOG00000003120	-1.14	-3.33	-6.27
ENSRNOG00000013223	-1.22	-2.78	-3.29
ENSRNOG00000012906	-1.19	-7.03	-12.26
ENSRNOG00000037957	-1.09	-1.11	-1.24
ENSRNOG00000017893	-1.56	-2.05	-2.17
ENSRNOG00000015035	-1.70	-9.18	-9.49

ENSRNOG00000006570	-1.23	-2.45	-4.67
ENSRNOG00000003183	-1.16	-2.56	-4.08
ENSRNOG00000010934	-1.87	-2.39	-1.94
ENSRNOG00000000307	-1.82	-2.07	-1.81
ENSRNOG00000023077	-1.80	-2.35	-1.78
ENSRNOG00000003650	-1.80	-2.11	-1.54
ENSRNOG00000003553	-1.40	-4.13	-5.49
ENSRNOG00000007922	-2.28	-3.80	-4.46
ENSRNOG00000024577	-1.02	-2.22	-2.13
ENSRNOG00000017414	-1.84	-2.60	-2.03
ENSRNOG00000005190	-2.61	-2.43	-1.43
ENSRNOG00000013656	-1.52	-6.38	-7.60
ENSRNOG00000038480	-1.74	-2.98	-2.11
ENSRNOG00000017628	-1.14	-1.80	-1.98
ENSRNOG00000002937	-2.10	-3.90	-6.22
ENSRNOG00000018476	-2.23	-3.48	-2.83
ENSRNOG00000008680	-1.42	-2.22	-3.62
ENSRNOG00000009531	-1.51	-2.78	-3.15
ENSRNOG00000024082	-4.79	-3.64	-3.68
ENSRNOG00000019057	-1.21	-3.74	-6.02
ENSRNOG00000026124	-2.18	-3.44	-2.59
ENSRNOG00000047046	-1.76	-7.75	-3.10
ENSRNOG00000017550	-2.24	-1.57	-1.80
ENSRNOG00000047860	-1.65	-4.28	-3.93
ENSRNOG00000011201	-2.30	-5.62	-4.61
ENSRNOG00000061768	-2.53	-2.75	-2.03

Table S8. The results of GO biological process enrichment.

Term Description	Gene Symbols	LogP	Rich Factor
oligodendrocyte development	Cd9,Mag,Kcnq3,Sox11,Lpar1,Zfp488,Myrf,Fa2h,Nkx6-2	-7.08	0.14
ensheathment of neurons	Cd9,Mag,Pllp,Prx,Cldn11,Lpar1,Bcas1,Myrf,Cmtm8,Fa2h,Nkx6-2,Gal3st1	-6.29	0.08
axon ensheathment	Cd9,Mag,Pllp,Prx,Cldn11,Lpar1,Bcas1,Myrf,Cmtm8,Fa2h,Nkx6-2,Gal3st1	-6.29	0.08
oligodendrocyte differentiation	Cd9,Cnp,Mag,Kcnq3,Sox11,Lpar1,Zfp488,Myrf,Fa2h,Nkx6-2	-5.64	0.08
myelination	Cd9,Mag,Pllp,Prx,Lpar1,Bcas1,Myrf,Cmtm8,Fa2h,Nkx6-2,Gal3st1	-5.50	0.07
glial cell development	Cd9,Mag,Kcnq3,Prx,Sox11,Lpar1,Zfp488,Myrf,Fa2h,Nkx6-2	-4.64	0.07
respiratory system development	Id1,Bmp4,Mgp,Ass1,Vegfa,Sox11,Hps1,Pou2f1,Aard,Ctsz,Insc,Wdpcp,Rcn3	-3.81	0.04
organic hydroxy compound metabolic process	Grin2a,Ldha,Npr1,Tpi1,Alpl,Inhba,Fah,Pctp,Pcbd1,Cpq,Crhr2,Prkaa2,Dhrs4,Moxd1,Ebpl,Cyb5r2,Naaa	-3.72	0.03
glucose catabolic process	Ldha,Tpi1,Hk2,Pfkl	-3.68	0.17
response to ammonium ion	Grin2a,Ass1,Kcnq3	-3.66	0.30
glial cell differentiation	Cd9,Cnp,Mag,Kcnq3,Prx,Sox11,Lpar1,C1s,Zfp488,Myrf,Fa2h,Nkx6-2	-3.60	0.04
kidney development	Ren,Bmp4,Nid1,Id3,Ass1,Prom1,Slc2a5,Vegfa,Sox11,Cdkn1c,Cd34,Wdpcp,Fat4,Heyl	-3.44	0.04
lung vasculature development	Id1,Bmp4,Vegfa	-3.41	0.25
gliogenesis	Tspo,Cd9,Cnp,Mag,Kcnq3,Prx,Sox11,Lpar1,C1s,Zfp488,Myrf,Fa2h,Nkx6-2	-3.32	0.04
renal system development	Ren,Bmp4,Nid1,Id3,Ass1,Prom1,Slc2a5,Vegfa,Sox11,Cdkn1c,Cd34,Wdpcp,Fat4,Heyl	-3.30	0.04
regulation of bone resorption	Syk,Cd38,Tfrc,Vegfa,Siglec15	-3.29	0.09
regulation of endothelial cell differentiation	Id1,Bmp4,S1pr2,Vegfa,Atoh8	-3.25	0.09

central nervous system myelination	Mag,Myrf,Fa2h,Nkx6-2	-3.24	0.13
axon ensheathment in central nervous system	Mag,Myrf,Fa2h,Nkx6-2	-3.24	0.13
regulation of epithelial cell differentiation	Stat5a,Id1,Bmp4,S1pr2,Prom1,Vegfa,Cdkn1c,Fat4,Atoh8	-3.15	0.05
regulation of oligodendrocyte differentiation	Bmp4,Zfp488,Myrf,Nkx6-2,Dusp15	-3.14	0.09
hexose catabolic process	Ldha,Tpi1,Hk2,Pfkl	-2.99	0.11
biogenic amine metabolic process	Grin2a,Npr1,Maob,Crhr2,Moxd1,Naaa	-2.98	0.07
myelin maintenance	Prx,Myrf,Fa2h	-2.94	0.18
supramolecular fiber organization	Fbln5,P4ha1,Fmod,Crhr2,Shroom1,Mical1,Nin,Rhoj,Kif19,Lmod1,Pkp1,Kash5,Loxl1,Fam107a,Msrb2,Myo7b,Pgm5	-2.94	0.03
regulation of myelination	Ctsc,Mag,Zfp488,Myrf,Nkx6-2	-2.91	0.08
regulation of bone remodeling	Syk,Cd38,Tfrc,Vegfa,Siglec15	-2.91	0.08
response to steroid hormone	Tspo,Adm,Bmp4,Mgp,Alpl,Cd38,Ass1,Maob,Inhba,Gpx3,Crhr2,Stc1,Vegfa,Kdm3a,Fam107a	-2.91	0.03
regulation of homotypic cell–cell adhesion	Cd9,Syk,Lgals1,Prkcq	-2.90	0.11
regulation of glial cell differentiation	Bmp4,Mag,Zfp488,Myrf,Nkx6-2,Dusp15	-2.86	0.06
monosaccharide catabolic process	Ldha,Tpi1,Hk2,Pfkl	-2.86	0.10
lung morphogenesis	Id1,Bmp4,Sox11,Hps1,Ctsz	-2.82	0.07
glomerulus development	Bmp4,Nid1,Prom1,Cd34,Heyl	-2.80	0.07
negative regulation of fatty acid oxidation	Fmo1,Acacb,Plin5	-2.79	0.16
response to xenobiotic stimulus	Tspo,Grin2a,Ldha,Ren,Apod,Fmo1,Gstt1,Cd38,Ass1,Maob,Ak4,Kcnq3,Lg	-2.78	0.03

	als1,Gpx3,Tfrc,Prkaa2,Vegfa,Acacb,As3mt		
pyridine-containing compound metabolic process	Ldha,Tpi1,Hk2,Fmo1,Alpl,Pfkl,Acacb	-2.75	0.05
response to corticosteroids	Adm,Bmp4,Mgp,Alpl,Ass1,Maob,Inhba,Gpx3,Crhr2,Stc1,Vegfa,Fam107a	-2.75	0.03
anatomical structure maturation	Ren,Kcnq3,Vegfa,Cdkn1c,Septin4,Phospho1,Fat4,Gldn,C1ql1,Baiap3	-2.72	0.04
small molecule biosynthetic process	Tpi1,Syk,Fmo1,Gamt,Alox5,Ass1,Inhba,Pcbd1,Prkaa2,Acacb,Dctd,Moxd1,Fa2h,Kdm3a	-2.71	0.03
amine metabolic process	Grin2a,Npr1,Maob,Crhr2,Moxd1,Naaa	-2.71	0.06
phenol-containing compound metabolic process	Grin2a,Npr1,Fah,Cpq,Crhr2,Moxd1	-2.67	0.06
response to selenium ions	Gstt1,Maob,Gpx3	-2.66	0.14
response to glucocorticoids	Adm,Bmp4,Mgp,Alpl,Ass1,Maob,Gpx3,Crhr2,Stc1,Vegfa,Fam107a	-2.65	0.03
substantia nigra development	Ldha,Cnp,Maob,Mag	-2.63	0.09
developmental maturation	Ren,Kcnq3,Vegfa,Cdkn1c,Rab38,Septin4,Phospho1,Fat4,Gldn,C1ql1,Baiap3	-2.62	0.03
muscle structure development	Ldha,Cd9,Adm,Plagl1,Bmp4,Id3,Ass1,Lgals1,Crhr2,Itga7,Vegfa,Sox11,Pdlim2,Heyl,Barx2,Pgm5	-2.62	0.03
regulation of gliogenesis	Tspo,Bmp4,Mag,Sox11,Zfp488,Myrf,Nkx6-2	-2.62	0.05
lung development	Id1,Bmp4,Mgp,Vegfa,Sox11,Hps1,Aard,Ctsz,Insc,Rcn3	-2.60	0.04
respiratory tube development	Id1,Bmp4,Mgp,Vegfa,Sox11,Hps1,Aard,Ctsz,Insc,Rcn3	-2.57	0.04
positive regulation of transmembrane receptor protein serine/threonine kinase signaling pathway	Bmp4,Inhba,Sox11,Cdkn1c,Slc2a10,Atoh8	-2.57	0.05
neuron projection regeneration	Tspo,Adm,Apod,Mag	-2.49	0.08

carbohydrate metabolic process	Ldha,Tpi1,Hk2,Apod,Pfkl,Ggta1,Fuom,Lancl3,Chst5,Spata20,Siae,Glb1l2,Pgm5	-2.44	0.03
catecholamine metabolic process	Grin2a,Npr1,Crhr2,Moxd1	-2.43	0.08
catechol-containing compound metabolic process	Grin2a,Npr1,Crhr2,Moxd1	-2.43	0.08
skeletal muscle organ development	Ldha,Plagl1,Ass1,Crhr2,Itga7,Sox11,Heyl,Barx2	-2.36	0.04
response to nutrients	Tspo,Ldha,Gstt1,Alox5,Mgp,Alpl,Ass1,Tfrc,Stc1,Vegfa,Acacb	-2.36	0.03
regulation of platelet aggregation	Cd9,Syk,Prkcq	-2.35	0.11
lens morphogenesis in camera-type eye	Bmp4,Sox11,Pou2f1	-2.35	0.11
neuron maturation	Kcnq3,Cdkn1c,Gldn,C1ql1	-2.34	0.07
ventricular septum morphogenesis	Bmp4,Sox11,Heyl,Rbm15	-2.34	0.07
receptor internalization	Cd9,Adm,Syk,Ramp1,Tfrc	-2.33	0.06
cellular response to glucocorticoid stimulus	Bmp4,Ass1,Crhr2,Stc1,Vegfa,Fam107a	-2.31	0.05
regulation of tissue remodeling	Syk,Cd38,Tfrc,Vegfa,Siglec15	-2.31	0.06
positive regulation of myelination	Mag,Zfp488,Myrf	-2.30	0.11
positive regulation of nervous system process	Grin2a,Mag,Zfp488,Myrf	-2.28	0.07
positive regulation of glial cell differentiation	Mag,Zfp488,Myrf,Nkx6-2	-2.28	0.07
nephron development	Bmp4,Nid1,Prom1,Slc2a5,Cd34,Fat4,Heyl	-2.28	0.04
organic acid biosynthetic process	Syk,Fmo1,Gamt,Alox5,Ass1,Pcbd1,Prkaa2,Acacb,Fa2h	-2.27	0.03
nephron epithelium development	Bmp4,Prom1,Slc2a5,Cd34,Fat4,Heyl	-2.25	0.05
pyridine nucleotide metabolic process	Ldha,Tpi1,Hk2,Fmo1,Pfkl,Acacb	-2.23	0.05

nicotinamide nucleotide metabolic process	Ldha,Tpi1,Hk2,Fmo1,Pfkl,Acacb	-2.23	0.05
negative regulation of gliogenesis	Tspo,Bmp4,Sox11,Nkx6-2	-2.17	0.07
cellular response to xenobiotic stimulus	Ren,Fmo1,Gstt1,Kcnq3,Tfrc,Prkaa2,Vegfa,As3mt	-2.17	0.04
positive regulation of gliogenesis	Tspo,Mag,Zfp488,Myrf,Nkx6-2	-2.15	0.05
regulation of lipid metabolic process	Tspo,Stat5a,Apod,Fmo1,Lsr,Prkaa2,Sctr,Acacb,Rab38,Asxl3,Plin5	-2.14	0.03
actin cytoskeleton organization	S1pr2,Crhr2,Shroom1,Pdlim2,Cdc42bpg,Mical1,Cldn19,Rhoj,Lmod1,Kash5,Fam107a,Msrb2,Myo7b,Pgm5	-2.13	0.03
cellular response to corticosteroid stimulus	Bmp4,Ass1,Crhr2,Stc1,Vegfa,Fam107a	-2.12	0.04
positive regulation of oligodendrocyte differentiation	Zfp488,Myrf,Nkx6-2	-2.10	0.09
regulation of cell fate commitment	Bmp4,Glis1,Nkx6-2	-2.10	0.09
actin filament organization	Crhr2,Shroom1,Mical1,Rhoj,Lmod1,Kash5,Fam107a,Msrb2,Myo7b	-2.08	0.03
dopamine metabolic process	Grin2a,Npr1,Moxd1	-2.06	0.09
skeletal muscle tissue development	Ldha,Plagl1,Crhr2,Itga7,Sox11,Heyl,Barx2	-2.05	0.04
nucleobase-containing small molecule metabolic process	Ldha,Npr1,Tpi1,Hk2,Fmo1,Gamt,Cnp,Pfkl,Ak4,Acot1,Acacb,Dctd,Gmds,Nt5c	-2.02	0.03
response to manganese ions	Tspo,Grin2a,Tfrc	-1.99	0.08
monocarboxylic acid metabolic process	Ldha,Tpi1,Hk2,Gamt,Alox5,Pfkl,Fah,Acot1,Prkaa2,Acacb,Cryl1,Dsel,Fa2h,Naaa	-1.93	0.02
ADP metabolic process	Tpi1,Hk2,Pfkl,Ak4	-1.92	0.06
skeletal muscle cell differentiation	Plagl1,Sox11,Heyl,Barx2	-1.92	0.06
plasma membrane organization	Cd9,Prx,Myrf,Xkr4,Fa2h,Fat4	-1.90	0.04

neural nucleus development	Ldha,Cnp,Maob,Mag	-1.90	0.06
axon regeneration	Tspo,Apod,Mag	-1.90	0.08
morphogenesis of a branching epithelium	Adm,Bmp4,Mgp,Vegfa,Ctsz,Fat4,Rbm15	-1.85	0.03
positive regulation of SMAD protein signal transduction	Bmp4,Inhba,Atoh8	-1.84	0.07
carboxylic acid biosynthetic process	Syk,Gamt,Alox5,Ass1,Pcbd1,Prkaa2,Acacb,Fa2h	-1.82	0.03
cell maturation	Ren,Kcnq3,Vegfa,Cdkn1c,Septin4,Gldn,C1ql1	-1.82	0.03
endothelial cell proliferation	Bmp4,Vegfa,Cd34	-1.81	0.07
cellular response to steroid hormone stimulus	Bmp4,Ass1,Crhr2,Stc1,Vegfa,Kdm3a,Fam107a	-1.81	0.03
purine nucleoside diphosphate metabolic process	Tpi1,Hk2,Pfkl,Ak4	-1.80	0.05
purine ribonucleoside diphosphate metabolic process	Tpi1,Hk2,Pfkl,Ak4	-1.80	0.05
regulation of nervous system process	Grin2a,Ctsc,Mag,S1pr2,Zfp488,Myrf	-1.78	0.04
actin filament-based process	S1pr2,Crhr2,Shroom1,Pdlim2,Cdc42bpg,Mical1,Cldn19,Rhoj,Lmod1,Kash5,Fam107a,Msrb2,Myo7b,Pgm5	-1.77	0.02
negative regulation of fatty acid metabolic process	Fmo1,Acacb,Plin5	-1.76	0.07
negative regulation of lipid metabolic process	Apod,Fmo1,Acacb,Asxl3,Plin5	-1.74	0.04
hexose metabolic process	Ldha,Tpi1,Hk2,Apod,Pfkl,Fuom	-1.74	0.04
kidney epithelium development	Bmp4,Prom1,Slc2a5,Cd34,Fat4,Heyl	-1.74	0.04
negative regulation of neurogenesis	Tspo,Id1,Bmp4,Mag,Sox11,Nkx6-2	-1.74	0.04

nucleotide metabolic process	Ldha,Npr1,Tpi1,Hk2,Fmo1,Cnp,Pfkl,Ak4,Acot1,Acacb,Dctd,Nt5c	-1.73	0.02
morphogenesis of a branching structure	Adm,Bmp4,Mgp,Vegfa,Ctsz,Fat4,Rbm15	-1.73	0.03
nucleoside phosphate metabolic process	Ldha,Npr1,Tpi1,Hk2,Fmo1,Cnp,Pfkl,Ak4,Acot1,Acacb,Dctd,Nt5c	-1.70	0.02
branching morphogenesis of an epithelial tube	Bmp4,Mgp,Vegfa,Ctsz,Fat4,Rbm15	-1.70	0.04
outflow tract morphogenesis	Bmp4,Vegfa,Sox11,Heyl	-1.69	0.05
regulation of fatty acid oxidation	Fmo1,Acacb,Plin5	-1.68	0.06
muscle organ development	Ldha,Plagl1,Id3,Ass1,Crhr2,Itga7,Sox11,Heyl,Barx2	-1.67	0.03
regulation of platelet activation	Cd9,Syk,Prkcq	-1.66	0.06
positive regulation of cell development	Tspo,Stat5a,Syk,Bmp4,Mag,S1pr2,Vegfa,Sox11,Zfp488,Myrf,Nin,Nkx6-2,Zbtb1	-1.66	0.02
steroid metabolic process	Tspo,Adm,Inhba,Pctp,Prkaa2,Dhrs4,Ebpl,Cyb5r2	-1.65	0.03
ribonucleoside diphosphate metabolic process	Tpi1,Hk2,Pfkl,Ak4	-1.64	0.05
cardiac septum morphogenesis	Bmp4,Sox11,Heyl,Rbm15	-1.64	0.05
negative regulation of nervous system development	Tspo,Id1,Bmp4,Mag,Sox11,Nkx6-2	-1.64	0.03
nucleotide catabolic process	Tpi1,Hk2,Cnp,Pfkl,Nt5c	-1.63	0.04
smooth muscle cell differentiation	Adm,Bmp4,Vegfa	-1.61	0.06
pyruvate metabolic process	Ldha,Tpi1,Hk2,Pfkl	-1.60	0.04
inner ear receptor cell differentiation	Bmp4,Tecta,Wdpcp,Fat4	-1.60	0.04
ventricular septum development	Bmp4,Sox11,Heyl,Rbm15	-1.60	0.04
positive regulation of B-cell activation	Stat5a,Syk,Cd38,Tfrc	-1.56	0.04

organic hydroxy compound biosynthetic process	Inhba,Pcbd1,Crhr2,Prkaa2,Moxd1,Cyb5r2	-1.56	0.03
glucose metabolic process	Ldha,Tpi1,Hk2,Apod,Pfkl	-1.55	0.04
odontogenesis	Adm,Bmp4,Id3,Alpl,Inhba	-1.55	0.04
nucleoside diphosphate metabolic process	Tpi1,Hk2,Pfkl,Ak4	-1.55	0.04
NAD metabolic process	Ldha,Tpi1,Pfkl	-1.54	0.06
nucleoside phosphate catabolic process	Tpi1,Hk2,Cnp,Pfkl,Nt5c	-1.54	0.04
tissue regeneration	Cd9,Apod,Cpq,Prkcq	-1.53	0.04
renal system process	Ren,Adm,Bmp4,Sctr,Cd34	-1.53	0.04
midbrain development	Ldha,Cnp,Maob,Mag	-1.52	0.04
regeneration	Tspo,Cd9,Adm,Apod,Mag,Cpq,Prkcq,Pou2f1	-1.52	0.03
cellular response to organic cyclic compound	Grin2a,Stat5a,Id1,Bmp4,Id3,Alpl,Ass1,Inhba,Lgals1,Crhr2,Stc1,Vegfa,Nrip1,Kdm3a,Fam107a	-1.52	0.02
purine-containing compound metabolic process	Ldha,Npr1,Tpi1,Hk2,Fmo1,Gamt,Pfkl,Ak4,Acot1,Acacb,Nt5c	-1.51	0.02
positive regulation of leukocyte activation	Stat5a,Syk,Ctsc,Cd38,Lgals1,Tfrc,Prkcq,Hps1,Zbtb1	-1.50	0.03
monosaccharide metabolic process	Ldha,Tpi1,Hk2,Apod,Pfkl,Fuom	-1.49	0.03
metanephros development	Bmp4,Id3,Slc2a5,Cd34	-1.48	0.04
mechanoreceptor differentiation	Bmp4,Tecta,Wdpcp,Fat4	-1.48	0.04
response to axon injury	Tspo,Apod,Mag,Lgals1	-1.45	0.04
muscle tissue development	Ldha,Plagl1,Bmp4,Crhr2,Itga7,Vegfa,Sox11,Heyl,Barx2,Pgm5	-1.44	0.02
regulation of nervous system development	Tspo,Id1,Bmp4,Ctsc,Mag,Vegfa,Sox11,Zfp488,Myrf,Nin,Flrt2,Nkx6-2,Heyl	-1.43	0.02

actin polymerization or depolymerization	Mical1,Fam107a,Msrb2	-1.43	0.05
nephron tubule development	Bmp4,Slc2a5,Fat4,Heyl	-1.40	0.04
glycolytic process	Tpi1,Hk2,Pfkl	-1.39	0.05
inner ear auditory receptor cell differentiation	Bmp4,Tecta,Wdpcp	-1.39	0.05
positive regulation of leukocyte cell–cell adhesion	Stat5a,Syk,Alox5,Lgals1,Tfrc,Prkcq,Zbtb1	-1.39	0.03
positive regulation of lymphocyte activation	Stat5a,Syk,Cd38,Lgals1,Tfrc,Prkcq,Hps1,Zbtb1	-1.38	0.03
positive regulation of ossification	Alox5,Bmp4,Sox11	-1.37	0.05
purine nucleotide metabolic process	Ldha,Npr1,Tpi1,Hk2,Fmo1,Pfkl,Ak4,Acot1,Acacb,Nt5c	-1.37	0.02
purine ribonucleotide catabolic process	Tpi1,Hk2,Pfkl,Nt5c	-1.37	0.04
regulation of leukocyte cell–cell adhesion	Stat5a,Syk,Alox5,Bmp4,Ass1,Lgals1,Tfrc,Prkcq,Zbtb1	-1.36	0.02
regulation of neurogenesis	Tspo,Id1,Bmp4,Mag,Vegfa,Sox11,Zfp488,Myrf,Nin,Nkx6-2,Heyl	-1.36	0.02
positive regulation of cell activation	Stat5a,Syk,Ctsc,Cd38,Lgals1,Tfrc,Prkcq,Hps1,Zbtb1	-1.36	0.02
regulation of cell–cell adhesion	Stat5a,Cd9,Syk,Alox5,Bmp4,Ass1,Lgals1,Tfrc,Vegfa,Prkcq,Zbtb1	-1.34	0.02
positive regulation of cell adhesion	Stat5a,Syk,Alox5,Nid1,Lgals1,Tfrc,Vegfa,Prkcq,Apbb1ip,Zbtb1,Plekha2	-1.34	0.02
positive regulation of lymphocyte proliferation	Stat5a,Syk,Cd38,Tfrc,Prkcq	-1.33	0.03
regulation of lymphocyte proliferation	Stat5a,Syk,Bmp4,Cd38,Tfrc,Sox11,Prkcq	-1.33	0.03
ADP catabolic process	Tpi1,Hk2,Pfkl	-1.32	0.05
renal tubule development	Bmp4,Slc2a5,Fat4,Heyl	-1.32	0.04

positive regulation of mononuclear cell proliferation	Stat5a,Syk,Cd38,Tfrc,Prkcq	-1.31	0.03
monocarboxylic acid biosynthetic process	Gamt,Alox5,Prkaa2,Acacb,Fa2h	-1.31	0.03
pyridine nucleotide catabolic process	Tpi1,Hk2,Pfkl	-1.30	0.05

Table S9. The result of KEGG enrichment.

Term Description	Gene Symbols	LogP	Rich Factor
Fructose and mannose metabolism	Tpi1,Hk2,Pfkl,Gmds	-2.99	0.11
TGF-beta signaling pathway	Id1,Bmp4,Id3,Inhba,Fmod,Tfrc	-2.57	0.05
Arginine and proline metabolism	Gamt,Maob,P4ha1,Cndp1	-2.31	0.07
Propanoate metabolism	Ldha,Acacb,Acss3	-2.14	0.09
Glycolysis / Gluconeogenesis	Ldha,Tpi1,Hk2,Pfkl	-1.66	0.05
Amino sugar and nucleotide sugar metabolism	Hk2,Gmds,Cyb5r2	-1.57	0.06
Hematopoietic cell lineage	Cd9,Cd38,Tfrc,Cd34	-1.55	0.04
Apoptosis	Cyct,Ctsc,Ctsz,Septin4,Traf1	-1.49	0.04
Fluid shear stress and atherosclerosis	Gstt1,Bmp4,Ass1,Prkaa2,Vegfa	-1.35	0.03
Glucagon signaling pathway	Ldha,Pfkl,Prkaa2,Acacb	-1.34	0.04

Table S10. The ATP content of neurons after 48 h treatment of different concentrations of Ni^{2+} (n=4).

Ni^{2+} (μM)	0	100	200	500
ATP content (%)	95.31	86.55	70.41	6.46
	102.17	88.97	70.44	5.05
	109.68	99.27	68.17	6.01
	92.85	96.31	73.45	5.76
Mean	100.00	92.77	70.62	5.82
Std.	7.56	6.00	2.17	0.59

Table S11. The relative gene expression of *Hk2*, *Ldha*, *Cd9*, and *Nfasc* after 48 h of treatment with different concentrations of Ni^{2+} (n=3).

Gene	Group	Ct	Expression	Mean Expression	Std.
Gapdh (internal reference)	control	16.14			
		16.51			
		16.71			
	100 μM Ni^{2+}	15.67			
		15.71			
		15.6			
	200 μM Ni^{2+}	15.09			
		15.1			
		15.22			
	500 μM Ni^{2+}	17.41			
		17.23			
		17.44			
Hk2	control	27.42	0.86	1.01	0.13
		27.08	1.09		
		27.11	1.07		
	100 μM Ni^{2+}	25.48	1.91	2.01	0.15
		25.28	2.19		
		25.45	1.95		
	200 μM Ni^{2+}	23.86	4.07	3.28	0.80
		24.17	3.29		
		24.58	2.47		
	500 μM Ni^{2+}	24.52	12.04	11.89	0.59
		24.48	12.38		
		24.62	11.24		
Ldha	control	16.65	0.80	1.01	0.19
		16.13	1.15		
		16.21	1.09		
	100 μM Ni^{2+}	14.8	1.67	1.68	0.02
		14.77	1.70		
		14.79	1.68		
	200 μM Ni^{2+}	13.16	3.61	3.36	0.24
		13.36	3.15		
		13.28	3.32		
	500 μM Ni^{2+}	15.04	4.58	4.44	0.20
		15.16	4.22		
		15.06	4.52		

Cd9	control	17.57	1.01	1.00	0.01
		17.59	1.00		
		17.61	0.99		
	100 μM Ni^{2+}	18.73	0.26	0.25	0.06
		18.51	0.30		
		19.19	0.19		
	200 μM Ni^{2+}	22.23	0.02	0.02	0.00
		22.48	0.01		
		22.26	0.02		
	500 μM Ni^{2+}	22.99	0.04	0.06	0.01
		22.46	0.06		
		22.39	0.07		
Nfasc	control	21.23	1.06	1.00	0.05
		21.37	0.97		
		21.36	0.97		
	100 μM Ni^{2+}	21.47	0.52	0.45	0.08
		21.96	0.37		
		21.63	0.47		
	200 μM Ni^{2+}	21.51	0.35	0.33	0.02
		21.59	0.33		
		21.69	0.31		
	500 μM Ni^{2+}	23.89	0.32	0.31	0.02
		23.86	0.32		
		24.05	0.28		

Table S12. A comparison between the effects of Ni^{2+} on gene expression in L929 cells and neocortical neurons.

Gene Symbol	L929 Cells			Neocortical Neurons			Gene Function	Pathway
	Expression	Ni^{2+} concentration (μM) (100, 200)	Ni^{2+} treatment time (h) (12, 24, 48, 72)	Expression	Ni^{2+} concentration (μM) (100, 200, 500)	Ni^{2+} treatment time (h) (48)		
Esd	up	200	≥24	normal	≥100	48	Serine-type peptidase and hydrolase.	
Cap2	up	200	≥24	normal	≥100	48	Actin binding.	
Prps2	up	200	≥24	normal	≥100	48	Nucleotide synthesis.	
Itsn1	up	200	≥24	normal	≥100	48	Coordinates endocytic membrane traffic with the actin assembly machinery. Plays a role in synaptic vesicle endocytosis in brain neurons.	
Cdc5l	down	200	≥24	normal	≥100	48	A positive regulator of cell cycle G2/M progression.	
Srsf6	down	200	≥24	normal	≥100	48	Nucleic acid binding and RNA binding.	
Cpsf2	down	200	≥24	normal	≥100	48	RNA binding.	
Myg1	down	200	≥24	normal	≥100	48	Mitochondrial RNA metabolic process and rRNA and mRNA processing.	
Ndufa2	down	100	≥12	normal	≥100	48	NADH:Ubiquinone Oxidoreductase Subunit A2, a subunit of the first enzyme complex in the electron transport chain located in the inner mitochondrial membrane.	electron transport chain pathway
Ndufa7	down	100	≥12	normal	≥100	48	NADH:Ubiquinone Oxidoreductase Subunit A7.	
Ndufb2	down	100	≥12	normal	≥100	48	NADH:Ubiquinone Oxidoreductase Subunit B2.	
Ndufa10	down	100	≥48	normal	≥100	48	NADH:Ubiquinone Oxidoreductase Subunit A10.	

Sdhb	down	100	≥12	normal	≥100	48	Encodes the iron–sulfur protein subunit of the succinate dehydrogenase (SDH) enzyme complex which plays a critical role in the mitochondria. This enzyme complex converts succinate to fumarate which releases electrons as part of the citric acid cycle, and the enzyme complex additionally provides an attachment site for the released electrons to be transferred to the oxidative phosphorylation pathway. The SDH enzyme complex plays a role in oxygen-related gene regulation through its conversion of succinate, which is an oxygen sensor that stabilizes the hypoxia-inducible factor 1 (HIF1) transcription factor.	electron transport chain pathway
Uqcrb	down	100	≥12	normal	≥100	48	This gene encodes a subunit of the ubiquinol-cytochrome c oxidoreductase complex. The protein encoded by this gene binds ubiquinone and participates in the transfer of electrons when ubiquinone is bound. This protein plays an important role in hypoxia-induced angiogenesis through mitochondrial reactive oxygen species-mediated signaling.	
Cox6a1	down	100	≥12	normal	≥100	48	Cytochrome C Oxidase Subunit 6A1. Cytochrome c oxidase (COX), the terminal enzyme of the mitochondrial respiratory chain, catalyzes the electron transfer from reduced cytochrome c to oxygen.	
Cox6b1	down	200	≥12	normal	≥100	48	Cytochrome C Oxidase Subunit 6B1.	
Cox6c	down	100	≥12	normal	≥100	48	Cytochrome C Oxidase Subunit 6C.	
Cox7a2	down	100	≥12	normal	≥100	48	Cytochrome C Oxidase Subunit 7A2.	
Cox7a2l	down	100	≥12	normal	≥100	48	Cytochrome C Oxidase Subunit 7A2 Like.	

Cox8a	down	100	≥12	normal	≥100	48	Cytochrome C Oxidase Subunit 8A.	electron transport chain pathway
Col4a2	up	200	≥12	normal	≥100	48	This gene encodes one of the six subunits of type IV collagen (Collagen Type IV Alpha 2 Chain), the major structural component of glomerular basement membranes (GBMs). Canstatin, a cleavage product corresponding to the collagen alpha 2(IV) NC1 domain, possesses both anti-angiogenic and anti-tumor cell activity. It inhibits the proliferation and migration of endothelial cells, reduces mitochondrial membrane potential, and induces apoptosis.	focal adhesion pathway
Col5a1	up	100	72	normal	≥100	48	This gene encodes an alpha chain (Collagen Type V Alpha 1 Chain) for one of the low abundance fibrillar collagens. It is a minor connective tissue component of nearly ubiquitous distribution. Type V collagen binds to DNA, heparan sulfate, thrombospondin, heparin, and insulin.	
Fn1	down	100	≥12	normal	≥100	48	This gene encodes fibronectin, a glycoprotein present in a soluble dimeric form in plasma, and in a dimeric or multimeric form at the cell surface and in the extracellular matrix. The encoded preproprotein is proteolytically processed to generate the mature protein. Fibronectin is involved in the cell adhesion and migration processes including embryogenesis, wound healing, blood coagulation, host defense, and metastasis.	

Vtn	down	200	≥12	normal	≥100	48	The protein encoded by this gene (Vitronectin) functions in part as an adhesive glycoprotein. The differential expression of this protein can promote either cell adhesion or migration as it links cells to the extracellular matrix through a variety of ligands. This protein also inhibits the membrane-damaging effect of the terminal cytolytic complement pathway and binds to several serpin serine protease inhibitors.	focal adhesion pathway
Igf1r	up	100	≥48	normal	≥100	48	This receptor binds insulin-like growth factor with a high affinity. It has tyrosine kinase activity. The insulin-like growth factor I receptor plays a critical role in transformation events. It is highly overexpressed in most malignant tissues where it functions as an anti-apoptotic agent by enhancing cell survival.	
Pten	up	200	≥12	normal	≥100	48	The protein encoded by this gene is a phosphatidylinositol-3,4,5-trisphosphate 3-phosphatase. It contains a tensin-like domain as well as a catalytic domain similar to that of dual specificity protein tyrosine phosphatases. Unlike most of the protein tyrosine phosphatases, this protein preferentially dephosphorylates phosphoinositide substrates. It negatively regulates intracellular levels of phosphatidylinositol-3,4,5-trisphosphate in cells and functions as a tumor suppressor by negatively regulating the AKT/PKB signaling pathway.	

Crk	up	100	72	normal	≥100	48	This gene encodes a member of an adapter protein family that binds to several tyrosine-phosphorylated proteins. It is involved in cell branching and adhesion mediated by BCAR1-CRK-RAPGEF1 signaling and the activation of RAP1.	focal adhesion pathway
Cdc42	up	100	≥12	normal	≥100	48	The protein encoded by this gene (Cell Division Cycle 42) is a small GTPase of the Rho subfamily, which regulates the signaling pathways that control diverse cellular functions including cell morphology, migration, endocytosis, and cell cycle progression.	
Rap1b	up	100	≥12	normal	≥100	48	This gene encodes a member of the RAS-like small GTP-binding protein superfamily. Members of this family regulate multiple cellular processes including cell adhesion and growth and differentiation.	
Pak6	up	100	≥12	normal	≥100	48	This gene encodes a member of a family of p21-stimulated serine/threonine protein kinases, which contain an amino-terminal Cdc42/Rac interactive binding (CRIB) domain and a carboxyl-terminal kinase domain. These kinases function in a number of cellular processes, including cytoskeleton rearrangement, apoptosis, and the mitogen-activated protein (MAP) kinase signaling pathway.	

Bad	up	100	≥12	normal	≥100	48	The protein encoded by this gene is a member of the BCL-2 family. BCL-2 family members are known to be regulators of programmed cell death. This protein positively regulates cell apoptosis by forming heterodimers with BCL-xL (extra-large B-cell lymphoma) and BCL-2, and reversing their death repressor activity. The proapoptotic activity of this protein is regulated through its phosphorylation. Protein kinases AKT and MAP kinase, as well as protein phosphatase calcineurin, were found to be involved in the regulation of this protein.	
Actb	down	100	72	normal	≥100	48	This gene encodes one of six different actin proteins (Actin Beta). Actins are highly conserved proteins that are involved in cell motility, structure, integrity, and intercellular signaling. The encoded protein is a major constituent of the contractile apparatus and one of the two nonmuscle cytoskeletal actins that are ubiquitously expressed.	focal adhesion pathway
Actg1	down	100	72	normal	≥100	48	Actin Gamma 1. Actins are highly conserved proteins that are involved in various types of cell motility and in the maintenance of the cytoskeleton. Three main groups of actin isoforms have been identified in vertebrate animals: alpha, beta, and gamma. The alpha actins are found in muscle tissues and are a major constituent of the contractile apparatus. The beta and gamma actins co-exist in most cell types as components of the cytoskeleton and as mediators of internal cell motility. Actin gamma 1, encoded by this gene, is a cytoplasmic actin found in all cell types.	

Mapk9	down	100	72	normal	≥100	48	The protein encoded by this gene (Mitogen-Activated Protein Kinase 9) is a member of the MAP kinase family. MAP kinases act as an integration point for multiple biochemical signals, and are involved in a wide variety of cellular processes such as proliferation, differentiation, transcription regulation, and development. This kinase targets specific transcription factors, and thus mediates immediate–early gene expression in response to various cell stimuli. This gene and MAPK8 are also known as c-Jun N-terminal kinases. This kinase blocks the ubiquitination of tumor suppressor p53, and thus increases the stability of p53 in nonstressed cells.	focal adhesion pathway
Bub3	down	100	72	normal	≥100	48	This gene encodes a protein involved in spindle checkpoint function. It has a dual function in spindle-assembly checkpoint signaling and in promoting the establishment of correct kinetochore–microtubule (K-MT) attachments. It promotes the formation of stable end-on bipolar attachments and is necessary for kinetochore localization of BUB1.	

Mcm6	down	100	72	normal	≥100	48	The protein encoded by this gene (Minichromosome Maintenance Complex Component 6) is one of the highly conserved mini-chromosome maintenance proteins (MCM) that are essential for the initiation of eukaryotic genome replication. The hexameric protein complex formed by MCM proteins is a key component of the pre-replication complex (pre_RC) and may be involved in the formation of replication forks and in the recruitment of other DNA replication-related proteins.	
Foxo3a	down	100	72	normal	≥100	48	This gene belongs to the forkhead family of transcription factors which are characterized by a distinct forkhead domain. This gene likely functions as a trigger for apoptosis through the expression of genes necessary for cell death.	
Hist1h1c	up	200	≥24	up	500	48	The histones H1 is necessary for the condensation of nucleosome chains into higher order structured fibers. It also acts as a regulator of individual gene transcription through chromatin remodeling, nucleosome spacing, and DNA methylation. Among its related pathways are cellular responses to stimuli and programmed cell death.	
Egln3	up	200	≥24	up	500	48	Enables peptidyl-proline 4-dioxygenase activity. Involved in several processes, including response to hypoxia; the activation of the cysteine-type endopeptidase activity involved in apoptotic process; and protein hydroxylation.	

Ndrg1	up	200	≥24	up	≥200	48	The protein encoded by this gene is a cytoplasmic protein involved in stress responses, hormone responses, cell growth, and differentiation. The encoded protein is necessary for p53-mediated caspase activation and apoptosis. It has a role in cell trafficking, notably of the Schwann cell, and is necessary for the maintenance and development of the peripheral nerve myelin sheath.	
Ier3	up	200	≥24	up	≥200	48	This gene functions in the protection of cells from Fas- or tumor necrosis factor type alpha-induced apoptosis.	
Tmpo	down	200	≥24	down	500	48	Through alternative splicing, this gene encodes several distinct LEM domains containing protein isoforms. LEM domain proteins include inner nuclear membrane and intranuclear proteins, and are involved in a variety of cellular functions including gene expression, chromatin organization, replication, and cell cycle control.	
Slc25a5	down	100	≥48	down	≥200	48	This gene is a member of the mitochondrial carrier subfamily of the solute carrier protein genes. The product of this gene functions as a gated pore that translocates ADP from the cytoplasm into the mitochondrial matrix and ATP from the mitochondrial matrix into the cytoplasm. The suppressed expression of this gene has been shown to induce apoptosis and inhibit tumor growth.	

Pik3cg	up	100	≥12	up	≥200	48	Generates PIP3, and PIP3 plays a key role by recruiting PH domain-containing proteins to the membrane, including AKT1 and PDPK1, activating the signaling cascades involved in cell growth, survival, proliferation, motility, and morphology. Links G-protein-coupled receptor activation to PIP3 production. Involved in immune, inflammatory, and allergic responses.	
Cdc20	down	100	72	down	≥200	48	CDC20 (Cell Division Cycle 20) appears to act as a regulatory protein interacting with several other proteins at multiple points in the cell cycle. Involved in the metaphase/anaphase transition of cell cycle. The CDC20-APC/C complex positively regulates the formation of synaptic vesicle clustering at active zone to the presynaptic membrane in postmitotic neurons. CDC20-APC/C-induced degradation of NEUROD2 induces presynaptic differentiation.	
